# NMR and DFT investigations of structure of colchicine in various solvents including density functional theory calculations

**DOI:** 10.1038/s41598-017-06005-5

**Published:** 2017-07-17

**Authors:** Gregory K. Pierens, T. K. Venkatachalam, David C. Reutens

**Affiliations:** The Centre for Advanced Imaging, The University of Queensland, Building 57, Research Road, St. Lucia, Queensland 4072 Australia

## Abstract

A detailed NMR investigation of the chemical shifts of hydrogen and carbon atoms associated with the structure of the naturally occurring alkaloid colchicine was conducted using high field NMR. Initially, the experimental chemical shifts for colchicine in chloroform and DMSO were compared to the values calculated using density functional theory (DFT). There were significant deviations observed for the chloroform solvent, but these were only slight in the DMSO solution. Dilution of the chloroform solution changed the experimental chemical shifts and improved agreement with the DFT calculations, suggesting self-aggregation at higher concentrations. A dimeric model was proposed for which agreement with the DFT calculated chemical shifts was better than for corresponding monomeric structures. Three further solvents were studied to evaluate changes in chemical shift values at different dilutions. Chloroform, benzene and water showed significant chemical shift changes implying self-aggregation, whereas DMSO and acetone did not show significant change upon dilution.

## Introduction

Colchicine is a naturally occurring alkaloid that binds to tubulin, inhibiting the formation of microtubules in mitotic spindles^1^ and suppressing cell division^2^. A number of clinical trials have been conducted utilizing colchicine as an anti-cancer drug, however clinical use is hampered by toxicity. Although nuclear magnetic resonance (NMR) investigations of colchicine have been conducted for nearly 30 years, there are several remaining discrepancies in the interpretation of the spectral data for this compound. In the first report on ^13^C NMR chemical shifts for colchicine by Singh *et al*.^3^, assignment of the individual carbon resonances was performed using structurally similar compounds such as tropolones. However, the low field strength of the spectrometer used in this study (60 MHz), necessitated the use of a large amount of compound and several resonances were superimposed, rendering assignment of the carbons difficult. Gaffied *et al*.^4^ used NMR techniques to ascertain the conformational isomerism of the structurally related colchicine analog, isocolchicine. The absolute configuration of natural colchicine was obtained in X-ray crystal studies by Brossi *et al*.^5^ (−) Colchicine was found to show a negative Cotton effect curve while (+) colchicine showed the opposite; this was ascribed to a skewed phenyl substituted tropolone structure^6^. Additional NMR studies were conducted by Mweksuriyen *et al*.^7^ to complete the ^13^C NMR assignment of the carbons in light of discrepancies in the earlier reports^8, 9^ despite a large literature regarding the structural aspects of colchicine^10–15^. The discrepancies associated with earlier studies can be ascribed due to the overlapping signals in the NMR spectrum as well as the lack of advanced pulse programs for detailed NMR analysis that are now available. For example the chemical shifts have been reported as being 150 ppm, 153.8 ppm and 151.1 ppm for C1 and 151.4 ppm and 153.5 ppm for C3^8, 9^. Recently, Virgili *et al*.^16^ conducted a detailed study on colchicine using multinuclear NMR. DFT calculations were performed to confirm the chemical shifts obtained experimentally but the calculated values for certain carbon atoms differed considerably from experimental values. The gas phase Gauge Independent Atomic Orbitals (GIAO) calculated carbon chemical shifts for several carbons (C1, C4, C6, C18 and C9) also differed from experimental values. These discrepancies prompted us to re-examine the proton and carbon chemical shifts for colchicine using high field NMR (700 MHz). This manuscript reports the assignments of proton and carbon chemical shifts of colchicine in several solvents. To resolve the discrepancies in the literature, we aimed to determine whether the polarity of solvents affected observed chemical shifts for individual carbons in the structure of colchicine. Additionally, we examined whether the concentration of colchicine in particular solvents affected chemical shift values.

## Experimental

All chemicals were purchased from Sigma-Aldrich and used without further purification. “Concentrated” samples were prepared as ∼46 mM of colchicine for all solvents used in this study. Additional samples were either diluted 100 fold from the ∼46 mM solution or prepared fresh as a ~0.46 mM solution. All NMR samples had volumes of 0.6 ml. For experiments using CDCl_3_, the solvent was exposed to basic alumina before preparing the samples to eliminate acidity from the solvent.

NMR data were acquired on a 700 MHz Bruker Avance III HD spectrometer equipped with a TCI cryoprobe. Here chemical shifts are reported in parts per million relative to the respective residual solvent peak as an internal standard. For the 46 mM solutions, the ^1^H NMR sweep width was 9 ppm and the number of scans was 32; the ^13^C NMR sweep width was 200 ppm and the number of scans was 256. Full structural elucidation was undertaken using ^1^H and ^13^C 1D NMR and a range of 2D NMR experiments (COSY, HSQC and HMBC). The number of scans was set at 2, 4 and 8 respectively for the COSY HSQC and HMBC datasets. The number of increments in the indirect dimension was set at 256 for all 2D experiments. All experiments were set up using th*e prosol* table in TOPSPIN 3.2. For the dilute samples, the scans were adjusted to give adequate signal to noise. The DOSY experiments were acquired with the *ledbpgp2s* pulse sequence with δ set to 4 ms and Δ set to 100 ms. Twenty four different gradient steps were used ranging from 2–95% with linear spacing. A recycle delay of 4 seconds was used, which included the acquisition time. The number of scans was set to 8 and 32 for the 46 mM and 0.46 mM samples.

### Molecular modeling and DFT calculations

Monte Carlo Conformational searching was performed using Macromodel (Schrodinger, LLC, New York, New York, USA)^17^. Torsional sampling using a Monte Carlo Multiple Minimum (MCMM) search was performed with 1000 steps per rotatable bond. Each step was minimized with the OPLS-2005 force field using the Truncated Newton Conjugate Gradient (TNCG) method with maximum iterations of 50,000 and energy convergence threshold of 0.02. All other parameters were left as the default values. The lowest energy conformations (<5 kcal/mol, 50 conformations) were optimized in Gaussian^18^.

All conformers were optimized with Gaussian 09^18^ using B3LYP/631 G(d) in vacuum and the vibrational frequencies where checked for a true minimum, i.e. no negative frequencies. All true minima were compared to remove identical structures or conformations which where <1% of the Boltzmann population. This resulted in 8 unique conformers for colchicine. The 8 unique conformations were further optimized with B3LYP/6311 + G(2d,p) and a Polarizable Continuum Model (PCM) for chloroform or DMSO and the vibrational frequencies where checked again for a true minimum. The free energies from the B3LYP/6311 + G(2d,p) calculation to calculate the Boltzmann Population and used to calculate the average chemical shifts.

NMR parameters (nmr = giao) were calculated with a single-point calculation, using two functional and basis set combinations; mpw1pw91/6-311 +G(2d,p) and B3LYP/6-311+G(2d,p)) using the optimized structures from the B3LYP/6-311 +G(2d,p) calculation. These two functional and basis set combinations were selected due to previously calculations giving good agreement between experimentally measured and calculated chemical shifts^19–21^. The integrated equation formalism polarized continuum model (IEFPCM)^22^ for chloroform or DMSO were used in all NMR calculations and the IEFPCM has been summarized and discussed elsewhere^23–25^. The computed NMR shielding tensors were converted to chemical shifts by the use of empirical scaling factors^23, 26^ that are derived from linear regression analysis of a test set of molecules^27^ at the same level of theory. The slope and intercept values of the scaling factors for mpw1pw91/6-311 + G(2d,p) and B3LYP/6-311 + G(2d,p) calculations are reported in the supporting information. Calculations for the dimer model used different functionals and basis sets, described later in this manuscript.

## Results and Discussion

**Figure 1:**
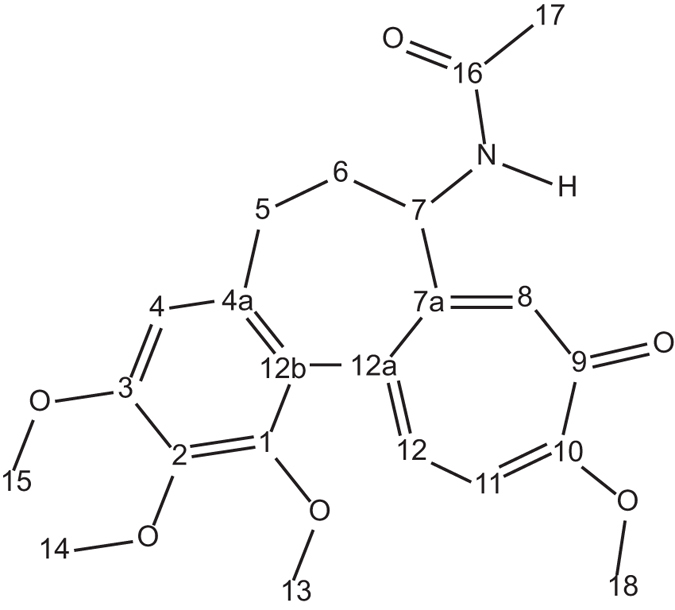
The numbering system used for colchicine (**1**) molecule for NMR analysis.

Solutions of colchicine in deuterated chloroform and DMSO were made up to ~46 mM concentration. The proton and carbon chemical shifts were confirmed by first principles using the ^1^H, ^13^C, COSY, HSQC and HMBC spectra. The chemical shifts were also calculated using density functional theory (DFT) and comparisons with experimentally measured chemical shifts are shown in Tables 1 and 2. The numbering of each of the atom in the colchicine molecule used in the present investigation is shown in Fig. 1.

**Table 1:** ^1^H Experimental and DFT calculated chemical shifts for colchicine in chloroform and DMSO solvents using two functional and basis set combinations.

Proton	Chloroform (46 mM)			DMSO (46 mM)		
	Measured	DFT Calc.^a^	DFT Calc.^b^	Measured	DFT Calc.^a^	DFT Calc.^b^
H_4	6.52	6.41	6.37	6.76	6.48	6.45
H_5	2.51	2.44	2.39	2.58	2.42	2.36
H_5	2.37	2.38	2.37	2.21	2.25	2.24
H_6	2.31	2.13	2.12	2.01	2.06	2.06
H_6	1.91	1.69	1.65	1.81	1.66	1.63
H_7	4.63	4.48	4.48	4.32	4.31	4.31
H_8	7.57	7.19	7.17	7.13	7.25	7.23
H_11	6.87	6.56	6.51	7.02	6.72	6.67
H_12	7.33	7.06	7.04	7.10	7.10	7.08
H_13	3.63	3.56	3.60	3.52	3.52	3.56
H_14	3.92	3.68	3.72	3.78	3.59	3.63
H_15	3.88	3.70	3.75	3.83	3.65	3.70
H_17	1.95	1.78	1.76	1.84	1.72	1.69
H_18	3.99	3.75	3.78	3.87	3.73	3.77
H_N	7.85	5.33	5.35	8.57	5.58	5.61
	MAE^c^	**0.19**	**0.19**	MAE	**0.13**	**0.13**
	Max. Dev.^c^	**0.38**	**0.40**	Max. Dev.	**0.30**	**0.35**

^a^Mpw1pw91/6-311 + g(2d,p) with IEPCM chloroform or dmso solvation. ^b^B3LYP/6-311 + g(2d,p) with IEPCM chloroform or dmso solvation. ^c^Does not include NH proton.

**Table 2:** ^13^C experimental and DFT calculated chemical shifts for colchicine in chloroform and DMSO solvents using two functional and basis set combinations.

^13^C	Chloroform (46 mM)			DMSO (46 mM)		
	Measured	DFT Calc.^a^	DFT Calc.^b^	Measured	DFT Calc.^a^	DFT Calc.^b^
C_1	151.2	150.4	151.1	150.5	148.9	149.6
C_2	141.6	140.5	141.3	140.7	139.1	140.0
C_3	153.6	152.7	153.4	152.9	151.8	152.5
C_4	107.3	104.4	103.9	107.7	104.5	104.0
C_4a	134.2	135.4	136.1	134.2	135.1	135.8
C_5	29.9	31.0	31.6	29.2	30.4	31.0
C_6	36.5	38.9	40.1	35.8	38.2	39.3
C_7	52.6	52.1	52.8	51.2	52.2	53.0
C_7a	152.3	150.6	151.1	150.8	150.4	150.8
C_8	130.5	130.9	130.4	130.4	129.6	129.1
C_9	179.5	174.5	174.4	178.0	173.8	173.7
C_10	163.8	164.2	164.8	163.5	163.0	163.4
C_11	112.8	109.0	108.6	112.1	109.5	109.2
C_12	135.6	136.6	136.4	134.4	136.2	135.9
C_12a	136.9	135.6	136.4	135.2	135.4	136.2
C_12b	125.6	126.4	127.1	125.4	124.9	125.6
C_13	61.6	58.5	58.6	60.7	58.0	58.1
C_14	61.4	58.0	58.2	60.8	57.9	58.1
C_15	56.1	52.9	53.1	55.8	52.9	53.2
C_16	170.1	166.4	166.7	168.5	166.7	166.9
C_17	22.8	21.9	21.4	22.4	21.8	21.4
C_18	56.4	53.3	53.5	56.0	53.3	53.5
	MAE	**1.94**	**1.93**	MAE	**1.71**	**1.76**
	Max. Dev.	**5.0**	**5.1**	Max. Dev.	**4.2**	**4.3**

^a^Mpw1pw91/6-311 + g(2d,p) with IEPCM chloroform or dmso solvation. ^b^B3LYP/6-311 + g(2d,p) with IEPCM chloroform or dmso solvation.

Two different functional and basis set combinations were used to calculate the DFT chemical shifts and it was found that the two methods (mpw1pw91/6-311 + g(2d,p) and B3LYP/6-311 + G(2d,p)) had very similar results for chloroform and DMSO solvents. From this point onwards the mpw1pw91/6-311 + G(2d,p) method will be discussed since it had the lowest maximum deviation between the experimentally measured and the DFT calculated chemical shifts.

The differences between measured proton NMR chemical shifts and DFT calculated values were larger in chloroform solvent with the largest difference being 2.55 ppm for the amide proton (Table 1). The next largest deviation was 0.38 ppm for H-8. Other large differences were observed for protons of the C ring and the methoxy group attached to position 10. The mean average deviation (MAE) for the chloroform comparison, calculated excluding the amide proton, was 0.19 ppm. The amide proton was not used in the calculation of the MAE since it is possibly in exchange with water or could be involved in hydrogen bonding interactions with itself or the solvent molecules and the chemical shift depends on strength of the hydrogen bond.

In DMSO, the maximum deviation was for the amide proton at 0.29 ppm, with H-11 and H-4 the next largest deviations at 0.30 and 0.28 ppm, respectively. The MAE for measurement made in DMSO was 0.13 ppm, also excluding the amide proton for the reasons given above. In both solvents a good agreement (within <0.2 ppm) between experimental and calculated chemical shifts was obtained but the DFT calculated MAE using DMSO had a slightly better fit for the whole molecule than that calculated for the chloroform solution. Interestingly, the upfield or downfield chemical shift changes seen in the experimental data associated with changing solvents were not always reflected by the DFT calculated chemical shifts. For example H-4 had a chemical shift of 6.52 ppm in chloroform and 6.76 ppm in DMSO and the DFT chemical shift showed a similar trend (6.41 ppm in chloroform and 6.48 ppm in DMSO). The DFT showed a smaller change in chemical shifts with solvent than the experimentally measured values. H-8 showed a chemical shift change from 7.57 with chloroform to 7.13 ppm with DMSO while the DFT showed a change in the opposite direction (7.19 with chloroform to 7.25 ppm with DMSO).

The carbon chemical shift data for chloroform and DMSO showed the largest changes for C-12a, C-16(C=O), C-9(C=O) and C-7a of >1.5 ppm. The largest changes in DFT calculated chemical shift values were for C-1, C-2, C-8, C-10 and C-12. It should be noted that the DFT calculations were for an isolated molecule using an implicit solvent model whereas the experimental measurements are made for compound surrounded by solvent molecules potentially with intermolecular interactions. This limitation highlights the caution required in using these types of computer models to calculate chemical shifts and in interpreting the results. Interestingly, the MAEs for both solvents were below 2 ppm (1.94 and 1.71 ppm for chloroform and DMSO respectively) which represents the data is in a good agreement.

Overall the calculated chemical shifts were in slightly better agreement with experimental measurements for DMSO. However, the large deviations for H-8 and C-9 in chloroform necessitated a detailed investigation. Two hypotheses were proposed to explain the observed deviations. The first hypothesis was that the chloroform solvent interacts with the carbonyl at C9 and the methoxy oxygen at C10. The second hypothesis is that colchicine molecules self-associate in solution at high concentrations. Self-association of colchicine has been observed by Chabin *et al*.^28^ in water using NMR spectroscopy.

To test both hypotheses, the chloroform solution was diluted approximately 100 times to a concentration of ~0.46 mM. If chloroform coordination was responsible for the observed differences, we would expect no change in the measured chemical shifts. On the other hand, colchicine self-association was predicted to change chemical shifts. DMSO was also diluted in the same way to test for concentration-related effects. Only ^1^H chemical shifts were investigated and are shown in Table 3.

**Table 3:** Comparison of ^1^H chemical shifts of colchicine in chloroform and DMSO at concentrations of 46 mM and 0.46 mM.

^1^H	Chloroform			DMSO		
	46 mM	0.46 mM	change	46 mM	0.46 mM	change
H_4	6.52	6.52	0.00	6.76	6.76	0.00
H_5	2.51	2.52	−0.01	2.58	2.52	0.06
H_5	2.37	2.41	−0.04	2.21	2.21	0.00
H_6	2.31	2.22	0.09	2.01	2.00	0.01
H_6	1.91	1.76	0.15	1.81	1.81	0.00
H_7	4.63	4.63	0.00	4.32	4.32	0.00
H_8	7.57	7.34	0.23	7.13	7.13	0.00
H_11	6.87	6.79	0.08	7.02	7.02	0.00
H_12	7.33	7.27	0.06	7.10	7.10	0.00
H_13	3.63	3.63	0.00	3.52	3.51	0.01
H_14	3.92	3.93	−0.01	3.78	3.78	0.00
H_15	3.88	3.89	−0.01	3.83	3.83	0.00
H_17	1.95	1.99	−0.04	1.84	1.84	0.00
H_18	3.99	3.98	0.01	3.87	3.87	0.00
H_N	7.85	5.89	1.96	8.57	8.55	0.02

As can be seen in Table 3, the chloroform solutions showed significant concentration effects but the DMSO solutions showed extremely minimal deviations. The largest chemical shift changes were observed for H6, H8 and NH protons in chloroform. The observed change for the NH proton chemical shift was very significant, from 7.85 ppm to 5.89 ppm upon dilution, i.e. a change of ~2 ppm. From the observed up field shift, we infer that the amide hydrogen (NH) is involved in a much stronger hydrogen bond at the higher concentration and upon dilution is involved in much weaker hydrogen bond at the lower concentrations. In contrast, for studies in DMSO solution, the NH proton chemical shift did not change and remained at ∼8.56 ppm. Therefore from these results we can conclude that colchicine in chloroform solution appear to self-aggregate at higher concentrations due to the concentration dependence of the proton chemical shifts, i.e. H6, H8 and NH. While in DMSO solution the molecule does not show any concentration dependence but the amide proton (NH) shows some evidence of interaction with the solvent.

Comparing the experimental and DFT calculated chemical shifts for the concentrated and diluted solutions of colchicine in chloroform, we observed that the diluted solution showed a reduction in the MAE from 0.19 to 0.15 ppm; the amide proton was not used in the calculation of this metric. From Table 4 we can see that the deviations between the experimental and calculated shifts was lower with dilution; for example the value for proton H8 decreased from 0.38 to 0.15 ppm. Based on these observations, we can eliminate the first hypothesis, in which chloroform solvent was coordinating with colchicine. Interestingly, the amide proton deviation decreased from 2.52 ppm to 0.56 ppm.

**Table 4:** Comparison of DFT calculated and experimentally measured chemical shifts at 46 mM and 0.46 mM.

^1^H	Chloroform (46 mM)			Chloroform (0.46 mM)		
	experimental	calculated	deviation	experimental	calculated	deviation
H_4	6.52	6.41	0.11	6.52	6.41	0.11
H_5	2.51	2.44	0.07	2.52	2.44	0.08
H_5	2.37	2.38	0.01	2.41	2.38	0.03
H_6	2.31	2.13	0.18	2.22	2.13	0.09
H_6	1.91	1.69	0.22	1.76	1.69	0.07
H_7	4.63	4.48	0.15	4.63	4.48	0.15
H_8	7.57	7.19	0.38	7.34	7.19	0.15
H_11	6.87	6.56	0.31	6.79	6.56	0.23
H_12	7.33	7.06	0.27	7.27	7.06	0.21
H_13	3.63	3.56	0.07	3.63	3.56	0.07
H_14	3.92	3.68	0.24	3.93	3.68	0.25
H_15	3.88	3.70	0.18	3.89	3.70	0.19
H_17	1.95	1.78	0.17	1.99	1.78	0.21
H_18	3.99	3.75	0.24	3.98	3.75	0.23
H_N	7.85	5.33	2.52	5.89	5.33	0.56
		MAE	**0.19**		MAE	**0.15**

**Figure 2:**
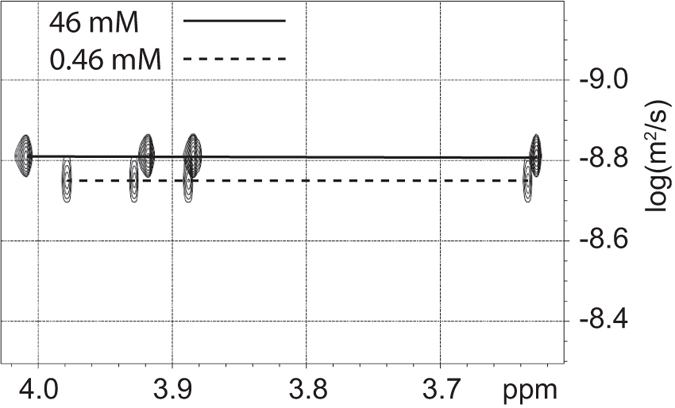
Overlay of the DOSY spectra for the 46 mM and 0.36 mM colchicine solutions in chloroform.

Diffusion Ordered Spectroscopy (DOSY) experiments were performed to examine differences in diffusion coefficients which would support the self-association hypothesis. Identical experiments were run on the 46 mM and the 0.46 mM samples in chloroform and DMSO solvents. A section of the 2D DOSY spectrum in chloroform is shown in Fig. 2. For chloroform, the higher concentration resulted in a diffusion coefficient of 1.54 × 10^−9^ m^2^/s compared to −1.77 × 10^−9^ m^2^/s (a change of 0.32 × 10^−9^ m^2^/s) for the diluted sample. In DMSO solvent (see supporting information), the diffusion coefficient changed by only 0.75 × 10^−10^ m^2^/s with dilution.

**Figure 3:**
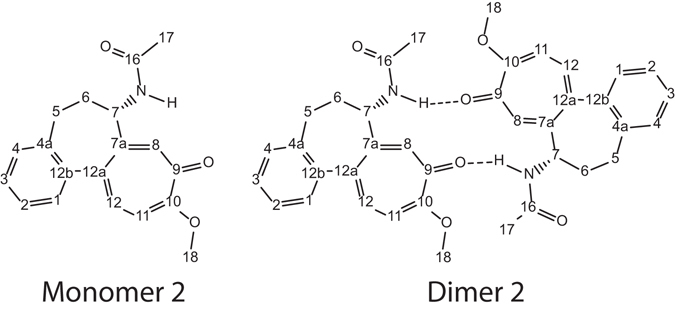
The partial structure of colchicine molecule used for DFT calculations.

As a consequence of the changes in chemical shifts and diffusion coefficient values with dilution in chloroform, a DFT calculation for a dimeric model of colchicine was performed. Using the information above, a cut-down version of colchicine (**2**) was used to reduce the overall calculation time since the largest changes observed were in the C ring. Hence, the three methoxy groups attached to the A ring of the colchicine molecule were removed. In using the cut down monomer **2** for calculation, we took the precaution of confirming that the chemical shifts of interest were not changed significantly from those of the parent colchicine molecule in the area of largest deviation in the experimentally measure chemical shifts between the concentrated and diluted solutions. The cut-down monomer **2** and dimer **2** molecules are shown in Fig. 3. We emphasize that this is a simplified dimer model as an aid to understanding the observed change in chemical shifts.

The chemical shift for the protons associated with ring C and other attached groups were compared between the full and the cut-down colchicine monomers (Table 5). The observed changes in ^1^H chemical shifts were not significant, supporting the use of the cut-down symmetric dimer for the purposes of this calculation. A symmetric dimer structure was selected since a single set of chemical shifts was observed in the NMR data. Two hydrogen bonds were utilized to hold the dimeric structure together, between the C9 carbonyl oxygen and the amide proton as shown in Fig. 3. This would allow the amide proton to be involved in a hydrogen bond, in keeping with its chemical shift change upon dilution, and preferentially places H8 between two aromatic rings.

**Table 5:** Comparison of key DFT calculated NMR chemical shifts between colchicine **1** and Monomer **2**.

^1^H	1	Monomer 2
H-7	4.48	4.44
H-8	7.19	7.26
H-11	6.56	6.57
H-12	7.06	7.08
H-17	1.78	1.81
H-18	3.75	3.73

Conformational searching of monomer **2** and Dimer **2** was undertaken using Macromodel^17^. For monomer **2** there were 5 conformers found and the DFT calculations using Gaussian^18^ were conducted as describe previously. After structure optimization removal of duplicate structures resulted in 3 conformations which were used in the DFT calculation of chemical shifts. For the dimer 2, a weak intramolecular hydrogen bond constraint (NH-CO distance of ~2 Å) and only one conformation was identified. Optimization with Gaussian^18^ was performed using wB97XD/6-311 + g(2d,p) since the functional includes empirical dispersion forces^29^. No constraints were imposed for the hydrogen bond. The DFT optimized structure resulted in an intramolecular NH–CO hydrogen bond that was 1.91 Å in length. The NMR chemical shifts were calculated for chloroform solution using three different functional and basis set combinations to calculate the NMR chemical shifts, B3LYP/6-311 + g(2d,p), mpw1pw91/6-311 + g(2d,p) and wB97XD/6-311 + g(2d,p); the last two are shown in Tables 6 and 7 (also see supporting information).

**Table 6:** Selected experimental and DFT calculated proton chemical shifts for Monomer 2 and Dimer 2 compared to the 46 mM colchicine in chloroform.

^1^H	Experimental	Calculated DFT chemical shifts (ppm)		
	46 mM	Monomer 2^a^	Dimer 2^a^	Dimer 2^b^
H-7	4.63	4.44	4.26	4.25
H-8	7.57	7.26	7.84	8.01
H-11	6.87	6.57	6.79	6.67
H-12	7.33	7.08	7.23	7.28
H-17	1.95	1.81	1.60	1.68
H-18	3.99	3.73	3.80	3.78
NH	7.85	5.35	9.22	9.32

^a^Calculated chemical shifts using mpw1pw91/6-311 + g(2d,p). ^b^Calculated chemical shifts using wB97XD/6-311 + g(2d,p).

**Table 7:** Selected experimental and DFT calculated proton chemical shifts for Monomer 2 and Dimer 2 compared to the 0.46 mM colchicine in chloroform.

1H	Experimental	Calculated DFT chemical shifts		
	0.46 mM	Monomer 2^a^	Dimer 2^a^	Dimer 2^b^
H-7	4.63	4.44	4.26	4.25
H-8	7.34	7.26	7.84	8.01
H-11	6.79	6.57	6.79	6.67
H-12	7.27	7.08	7.23	7.28
H-17	1.99	1.81	1.60	1.68
H-18	3.98	3.73	3.80	3.78
NH	5.35	5.35	9.22	9.32

^a^Calculated chemical shifts using mpw1pw91/6-311 + g(2d,p). ^b^Calculated chemical shifts using wB97XD/6-311 + g(2d,p).

The DFT calculated proton chemical shifts are shown in Table 6 for Monomer **2** and Dimer **2** for selected proton as compared to the experimental chemical shift of colchicine at 46 mM in chloroform. The results obtained from the two functional and basis set combinations for dimer **2** were very similar. The major differences were found for H-8, H-11 and the NH which varied by 0.17, 0.12 and 0.10 ppm respectively. The experimental chemical shift for H-8 in the 46 mM sample was 7.57 ppm which lies between the calculated values for the monomer and dimer models. This suggests that 46 mM colchicine in chloroform was neither entirely in monomer nor in dimer form in solution. This explanation was further supported by the observed NH chemical shift between the calculated value for the monomer and dimer. The other protons had lower calculated chemical shift values than those measured experimentally. Therefore from these results we conclude that colchicine in chloroform solution at concentration between 0.46 and 46 mM is a mixture of monomer and dimer.

In 1990, Chabin *et al*.^28^ reported that in water, protons in the A and C rings of colchicine moved up field with increasing concentration and inferred that both conjugated rings were involved in self-association or dimerization. This is different to our observations in chloroform solution where only the protons of C ring deviated significantly. Chabin *et al*.^28^ stated that the conformation of colchicine did not change with increasing concentration.

**Figure 4:**
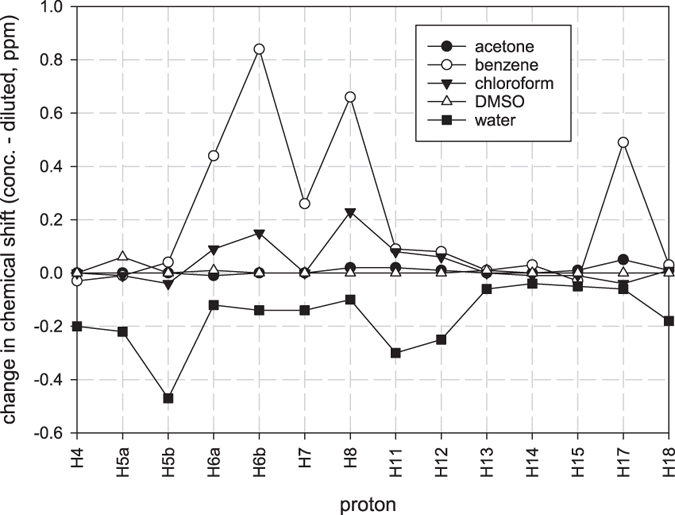
Differences observed in the proton chemical shift values for all the protons in various solvents of colchicine molecule.

We expanded our investigation of concentration-dependent effects by studying the proton NMR chemical shifts of several other deuterated solvents; acetone, benzene and D_2_O. Figure 4 shows the differences in chemical shifts between concentrated (~46 mM) and diluted samples (~0.46 mM) for all solvents used. Three distinct patterns emerged. Acetone and DMSO exhibited small differences between the two concentrations. Chloroform and benzene showed an up field shift upon dilution and water resulted in down field shifts with dilution, consistent with the values reported by Chabin *et al*.^28^.

The results obtained for acetone and DMSO solutions suggest that these solvents interfere with the formation of the dimer. In these solvents, the NH shift also did not change, implying that acetone and DMSO solvents themselves interacts with the amide proton, possibly forming a hydrogen bond since the shift remained at approximately 7.7 ppm. For chloroform and benzene, the major changes were observed mainly in the C ring, H6 and the acetamide group. Behavior in benzene and in chloroform solution appeared similar, pointing to self-aggregation in both solvents. In benzene solution, the amide proton also showed a large change with dilution (~3.4 ppm); in dilute solutions the amide proton is unlikely to be involved in a hydrogen bond. Changes with dilution were larger in benzene than in chloroform but both were in an up field direction.

For solutions in water, dilution resulted in a downfield shift and most of the protons affected are in A, B and C rings, as reported by Chabin *et al*.^28^. The largest changes observed were for H5, H11 and H12 protons. This may be explained by self-aggregation that is different from that in chloroform and benzene solutions since the H8 proton is not among the most affected chemical shifts.

In conclusion we have observed that colchicine appears to self-associate in solution in chloroform, benzene and water but not in DMSO or acetone. Self-association in benzene and chloroform seems to principally involve the C ring with the amide proton moving up field with dilution. In water it seems to involve A, B and C rings. The amide proton of colchicine in DMSO and acetone did not change in their chemical shift values with dilution, remaining >7.5 ppm, implying that they may be hydrogen bonded to the solvent.

## Electronic supplementary material

Supporting information
