# Supplementary material for: NMR and DFT investigations of structure of colchicine in various solvents including density functional theory calculations
Source: Sci Rep. 2017 Jul 17;7:5605. doi: 10.1038/s41598-017-06005-5 (PMC5514032; doi:10.1038/s41598-017-06005-5)
Supplement: Supplementary file 1 — Supporting information [file 41598_2017_6005_MOESM1_ESM.pdf]

NMR and DFT investigations of structure of colchicine in various solvents including Density functional theory calculations

Gregory K. Pierens[†], T.K. Venkatachalam,*[†] and David C. Reutens

Centre for Advanced Imaging, Building 57, Research Road,

The University of Queensland, St. Lucia, Queensland, 4072, Australia.

S1. Experimental ^{1}H chemical shifts for the ~46mM solutions.

S2. Experimental ^{1}H chemical shifts for the ~0.46 mM solutions.

S3. Experimental ^{13}C chemical shifts for the ~46mM solutions.

S4. Overlay of the DOSY spectra for the 46 mM and 0.46 mM colchicine solutions in chloroform and DMSO.

S5. Molecular modeling and DFT calculations.

S6. Scaling Factors used for converting the DFT calculated computed NMR shielding tensors into chemical shifts.

S7. Optimized coordinates for colchicine in chloroform solvent (B3LYP/6311+G(2d,p))

S8. Optimized coordinates for colchicine in DMSO solvent (B3LYP/6311+G(2d,p))

S9. Optimized coordinates for the cutdown colchicine (monomer 2) in chloroform solvent (B3LYP/6311+G(2d,p))

S10. Comparison of ^{1}H experimentally measured and DFT chemical shifts for colchicine (46mM) in chloroform using mpw1pw91/6311+G(2d,p) and B3LYP/6311+G(2d,p)

S11. Comparison of ^{1}H experimentally measured and DFT chemical shifts for colchicine (0.46mM) in chloroform using mpw1pw91/6311+G(2d,p) and B3LYP/6311+G(2d,p)

S12. Comparison of ^{13}C experimentally measured and DFT chemical shifts for colchicine in chloroform using mpw1pw91/6311+G(2d,p) and B3LYP/6311+G(2d,p)

S13. Comparison of ^{1}H experimentally measured and DFT chemical shifts for colchicine (46mM) in DMSO using mpw1pw91/6311+G(2d,p) and B3LYP/6311+G(2d,p)

S14. Comparison of ^{1}H experimentally measured and DFT chemical shifts for colchicine (0.46mM) inDMSO using mpw1pw91/6311+G(2d,p) and B3LYP/6311+G(2d,p)

S15. Comparison of ^{13}C experimentally measured and DFT chemical shifts for colchicine (46mM) using mpw1pw91/6311+G(2d,p) and B3LYP/6311+G(2d,p)

S16. Optimized coordinates for the dimer of the cutdown colchicine (dimer 2) in chloroform solvent (wB97XD/6-311+g(2d,p))

S17. Comparison of ^{1}H experimentally measured and DFT calculated chemical shifts for cut-down colchicine dimer 2.

S1. Experimental ^{1}H chemical shifts for the ~46mM solutions.

#	acetone	benzene	chloroform	DMSO	water
H_4	6.74	6.19	6.52	6.76	6.69
H_5	2.61	2.33	2.51	2.58	2.44
H_5	2.38	2.17	2.37	2.21	1.87
H_6	2.15	2.17	2.31	2.01	2.16
H_6	1.88	2.11	1.91	1.81	1.86
H_7	4.51	4.99	4.63	4.32	4.31
H_8	7.23	8.19	7.57	7.13	7.39
H_11	6.95	6.14	6.87	7.02	7.06
H_12	7.13	7.18	7.33	7.10	7.30
H_13	3.60	3.87	3.63	3.52	3.56
H_14	3.85	3.78	3.92	3.78	3.89
H_15	3.88	3.38	3.88	3.83	3.90
H_17	1.96	1.97	1.95	1.84	2.00
H_18	3.92	3.18	3.99	3.87	3.86
H_N	7.77	8.79	7.85	8.57	-

S2. Experimental ^{1}H chemical shifts for the ~0.46 mM solutions.

#	acetone	benzene	chloroform	DMSO	water
H_4	6.74	6.22	6.52	6.76	6.89
H_5	2.61	2.34	2.52	2.52	2.66
H_5	2.38	2.13	2.41	2.21	2.34
H_6	2.16	1.73	2.22	2.00	2.28
H_6	1.88	1.27	1.76	1.81	2.00
H_7	4.51	4.73	4.63	4.32	4.45
H_8	7.21	7.53	7.34	7.13	7.49
H_11	6.93	6.05	6.79	7.02	7.36
H_12	7.12	7.10	7.27	7.10	7.55
H_13	3.60	3.86	3.63	3.51	3.62
H_14	3.85	3.75	3.93	3.78	3.93
H_15	3.87	3.41	3.89	3.83	3.95
H_17	1.91	1.48	1.99	1.84	2.06
H_18	3.91	3.15	3.98	3.87	4.04
H_N	7.71	5.38	5.89	8.55	-

S3. Experimental ^{13}C chemical shifts for the ~46mM solutions.

#	acetone	benzene	chloroform	DMSO	water
C_1	151.5	152.5	151.2	150.5	149.8
C_2	142.6	143.0	141.6	140.7	140.0
C_3	154.6	154.6	153.6	152.9	153.1
C_4	108.6	108.2	107.3	107.7	108.1
C_4a	135.4	135.0	134.2	134.2	135.4
C_5	30.5	30.6	29.9	29.2	28.8
C_6	37.2	37.2	36.5	35.8	35.2
C_7	52.8	53.4	52.6	51.2	52.9
C_7a	151.5	153.6	152.3	150.8	153.4
C_8	131.7	131.9	130.5	130.4	129.6
C_9	179.4	180.1	179.5	178.0	179.7
C_10	165.1	165.1	163.8	163.5	164.3
C_11	112.5	113.1	112.8	112.1	115.3
C_12	135.2	135.7	135.6	134.4	137.4
C_12a	136.5	137.5	136.9	135.2	137.1
C_12b	127.1	127.0	125.6	125.4	124.7
C_13	61.5	61.4	61.6	60.7	61.4
C_14	61.3	62.1	61.4	60.8	61.6
C_15	56.4	56.0	56.1	55.8	56.0
C_16	169.4	169.6	170.1	168.5	173.8
C_17	22.9	23.1	22.8	22.4	21.6
C_18	56.4	55.8	56.4	56.0	56.3

S4. Overlay of the DOSY spectra for the 46 mM and 0.46 mM colchicine solutions in chloroform and DMSO.

Chloroform

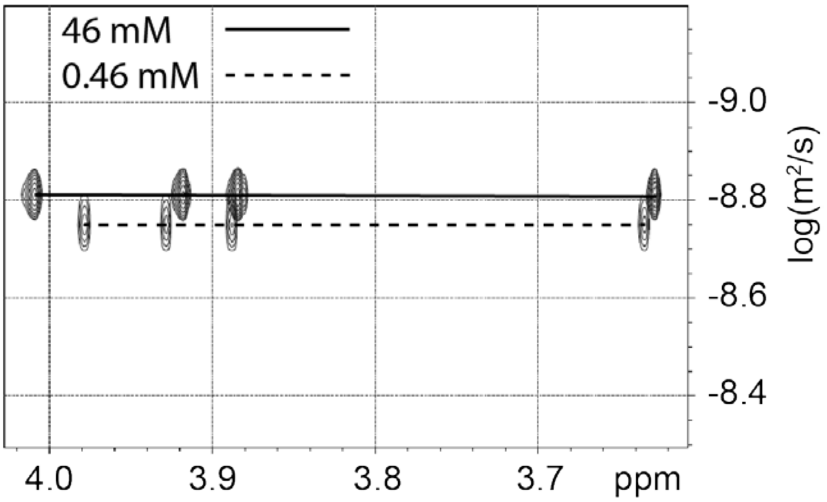

DMSO

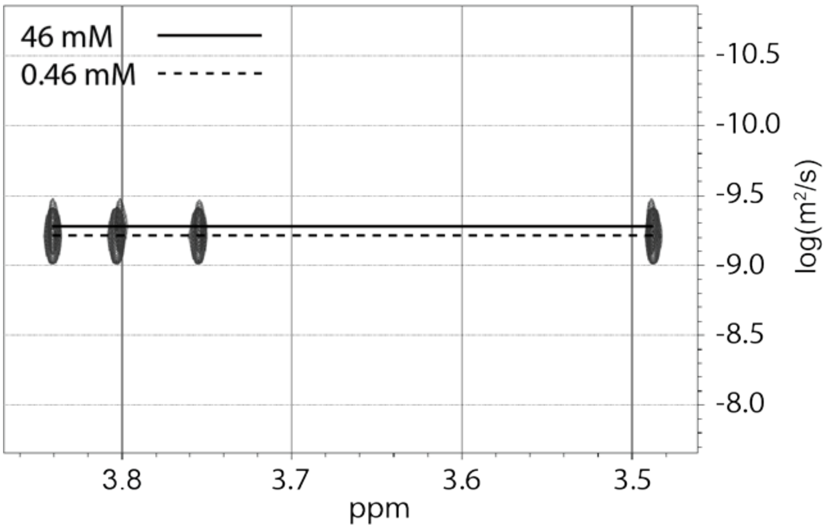

S5. Molecular modeling and DFT calculations.

Monte Carlo Conformational searching was performed using Macromodel (Schrodinger, LLC, New York, New York, USA). Torsional sampling using a Monte Carlo Multiple Minimum (MCMM) search was performed with 1000 steps per rotatable bond. Each step was minimized with the OPLS-2005 force field using the Truncated Newton Conjugate Gradient (TNCG) method with maximum iterations of 50,000 and energy convergence threshold of 0.02. All other parameters were left as the default values. The lowest energy conformations (< 5 kcal/mol, 50 conformations) were optimized in Gaussian.[18]

All conformers were optimized with Gaussian 09 using B3LYP/631G(d) in vacuum and the vibrational frequencies where checked for a true minimum, i.e. no negative frequencies. All true minima were compared to remove identical structures or conformations which where < 1% of the Boltzmann population. This resulted in 8 unique conformers for colchicine. The 8 unique conformations were further optimized with B3LYP/6311+G(2d,p) and a Polarizable Continuum Model (PCM) for chloroform or DMSO and the vibrational frequencies where checked again for a true minimum. The free energies from the B3LYP/6311+G(d,p) calculation to calculate the Boltzmann Population and used to calculate the average chemical shifts.

NMR parameters (nmr=giao) were calculated with a single-point calculation, using two functional and basis set combinations; mpw1pw91/6-311+G(2d,p) and B3LYP/6-311+G(2d,p)) using the optimized structures from the B3LYP/6-311+G(2d,p) calculation. The integrated equation formalism polarized continuum model (IEFPCM) for chloroform or DMSO were used in all NMR calculations The computed NMR shielding tensors were converted to chemical shifts by the approach of using empirical scaling factors that are derived from linear regression analysis of a test set of molecules at the same level of theory.

S6. Scaling Factors used for converting the DFT calculated computed NMR shielding tensors into chemical shifts.

Functional	Basis set	Solvent	Nucleus	slope	intercept
mpw1pw91	6-311+G(2d,p)	Chloroform	Proton	-1.072	31.873
mpw1pw91	6-311+G(2d,p)	Chloroform	Carbon	-1.042	186.357
mpw1pw91	6-311+G(2d,p)	DMSO	Proton	-1.060	31.722
mpw1pw91	6-311+G(2d,p)	DMSO	Carbon	-1.050	186.253
B3LYP	6-311+G(2d,p)	Chloroform	Proton	-1.056	31.934
B3LYP	6-311+G(2d,p)	Chloroform	Carbon	-1.043	181.717
B3LYP	6-311+G(2d,p)	DMSO	Proton	-1.042	31.784
B3LYP	6-311+G(2d,p)	DMSO	Carbon	-1.050	181.603
wB97XD	6-311+G(2d,p)	Chloroform	Proton	-1.056	31.934

S7. Optimized coordinates for colchicine in chloroform solvent (B3LYP/6311+G(2d,p))

Compound: Colchicine, Conformer: 01, Energy:-1359.468776 Hartree, Solvent: chloroform, Boltzmann %: 68.421

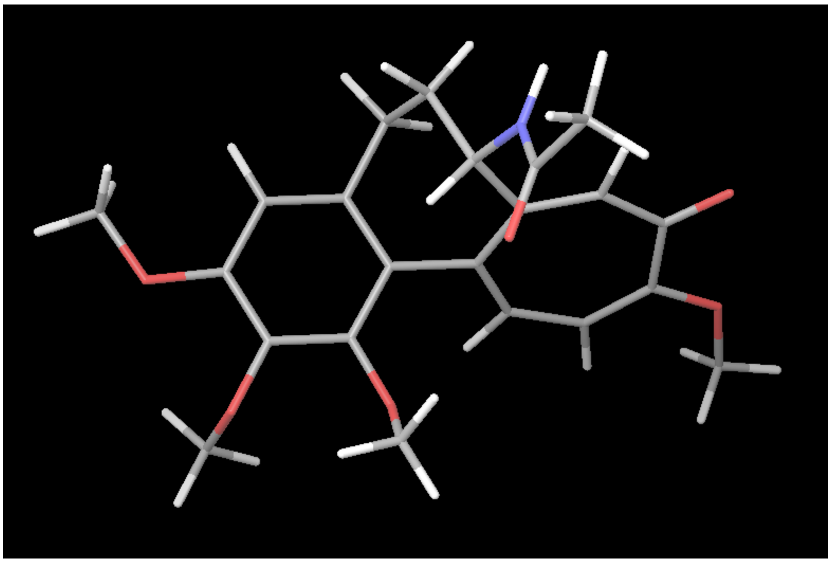

O	1.065144	2.802642	2.290820
C	1.648501	3.417809	1.404723
N	1.556881	3.068536	0.092939
C	0.734988	1.964692	-0.371430
C	1.436868	0.594219	-0.392499
C	2.801566	0.596073	-0.456669
C	3.835312	-0.414386	-0.526191
O	5.014516	-0.060457	-0.669945
C	3.527966	-1.854327	-0.401814
O	4.658832	-2.580066	-0.387853
C	4.600899	-3.999477	-0.256267
C	2.285235	-2.426102	-0.329393
C	0.994205	-1.851987	-0.341815
C	0.561314	-0.548369	-0.369492
C	-0.920434	-0.337409	-0.396581
C	-1.508458	0.444277	-1.403813

C	-2.891374	0.599019	-1.466227
C	-3.723091	-0.010987	-0.529878
O	-5.078955	0.082036	-0.520023
C	-5.709743	0.861259	-1.532697
C	-3.152866	-0.784755	0.492171
O	-3.946035	-1.336897	1.467088
C	-4.525949	-2.601886	1.120286
C	-1.766765	-0.943112	0.552396
O	-1.240912	-1.738101	1.541684
C	-1.160131	-1.119478	2.836353
C	-0.622707	1.154060	-2.398545
C	0.149738	2.318750	-1.752557
C	2.517244	4.619503	1.712766
H	-0.083638	1.885665	0.344079
H	2.061509	3.618011	-0.589655
H	3.267863	1.575218	-0.463397
H	4.078095	-4.452593	-1.102373
H	5.636369	-4.330701	-0.247853
H	4.114569	-4.289110	0.678613
H	2.263785	-3.507126	-0.273192
H	0.205703	-2.594382	-0.320722
H	-3.311179	1.203519	-2.258958
H	-6.776723	0.797620	-1.331693
H	-5.392002	1.906235	-1.483355
H	-5.501846	0.460678	-2.528716
H	-3.744883	-3.343751	0.934284
H	-5.167631	-2.505807	0.241457
H	-5.124305	-2.908792	1.976665
H	-0.730736	-1.866422	3.501823
H	-0.507659	-0.242936	2.800741
H	-2.152132	-0.835052	3.190504

H	0.089657	0.443390	-2.826530
H	-1.219661	1.537805	-3.227610
H	0.960270	2.624606	-2.423183
H	-0.509456	3.181443	-1.622531
H	1.921372	5.350857	2.260651
H	3.332681	4.302359	2.365156
H	2.937177	5.091945	0.824091

Compound: Colchicine, Conformer: 02, Energy:-1359.466803 Hartree, Solvent: chloroform, Boltzmann %: 8.466

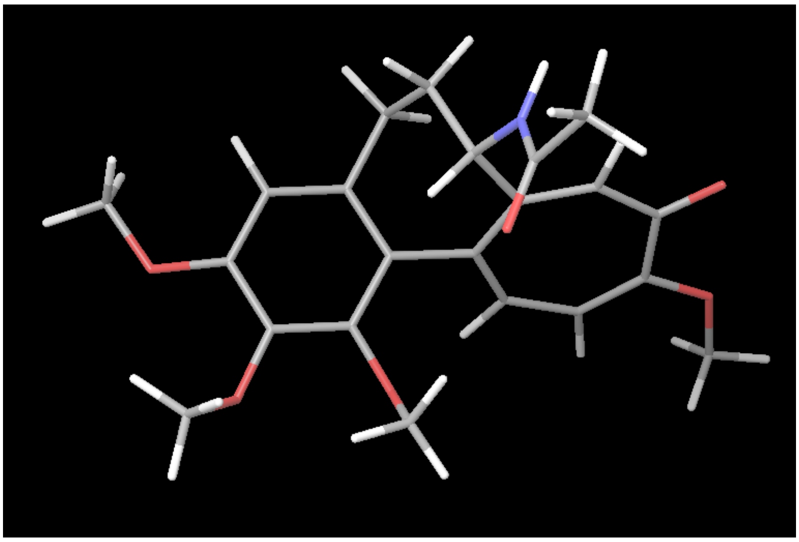

O	0.793609	2.873042	2.188591
C	1.386752	3.483156	1.305130
N	1.371337	3.082336	0.005124
C	0.631947	1.918342	-0.450685
C	1.417903	0.594573	-0.418271
C	2.781211	0.678966	-0.454764
C	3.875460	-0.268051	-0.475549
O	5.033202	0.152234	-0.614669
C	3.653929	-1.718943	-0.303721
O	4.826124	-2.371550	-0.230958
C	4.851118	-3.785692	-0.041892
C	2.447088	-2.364673	-0.246617
C	1.124340	-1.872507	-0.309723
C	0.614147	-0.598890	-0.382984
C	-0.876806	-0.480870	-0.449810
C	-1.484990	0.231556	-1.494226
C	-2.872657	0.275818	-1.601207
C	-3.686381	-0.372809	-0.675932
O	-5.044464	-0.422724	-0.748372
C	-5.691657	0.234413	-1.834761

C	-3.098665	-1.046434	0.408637
O	-3.848050	-1.731736	1.328361
C	-4.737276	-0.956563	2.146690
C	-1.704366	-1.106995	0.502169
O	-1.146591	-1.843184	1.519324
C	-1.000024	-1.151399	2.765613
C	-0.622102	0.972558	-2.486178
C	0.060311	2.199707	-1.854101
C	2.183786	4.735596	1.604813
H	-0.197977	1.806828	0.247008
H	1.878416	3.632014	-0.675600
H	3.187714	1.684115	-0.480313
H	4.385955	-4.304025	-0.884138
H	5.904129	-4.050554	0.012457
H	4.352204	-4.067600	0.888666
H	2.490304	-3.442761	-0.157038
H	0.381992	-2.660984	-0.287366
H	-3.312559	0.811233	-2.431752
H	-6.755522	0.057072	-1.694540
H	-5.496472	1.310110	-1.820606
H	-5.376198	-0.184041	-2.794213
H	-5.562089	-0.551315	1.561404
H	-4.197488	-0.140743	2.636073
H	-5.119076	-1.641992	2.901466
H	-0.503404	-1.844553	3.442472
H	-0.385713	-0.255208	2.643044
H	-1.973480	-0.876878	3.177455
H	0.143802	0.298509	-2.879706
H	-1.221870	1.296908	-3.338305
H	0.866585	2.538827	-2.513745
H	-0.653934	3.022625	-1.762510

H	1.534611	5.451333	2.111002
H	2.991386	4.478204	2.292446
H	2.610277	5.202726	0.716477

Compound: Colchicine, Conformer: 03, Energy:-1359.466726 Hartree, Solvent: chloroform, Boltzmann %: 7.803

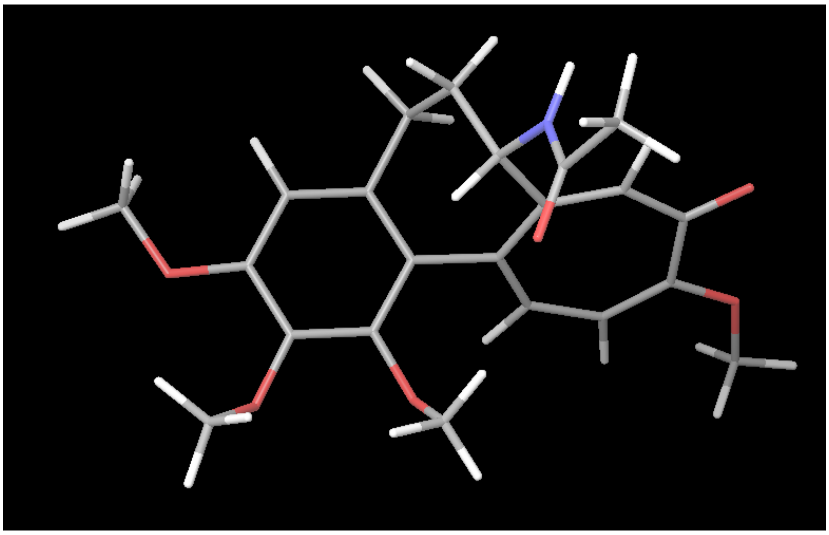

O	0.851353	2.816019	2.167909
C	1.407988	3.454298	1.280422
N	1.352949	3.081475	-0.026856
C	0.614261	1.914314	-0.474588
C	1.402522	0.592817	-0.435009
C	2.765142	0.676414	-0.486907
C	3.857553	-0.272815	-0.504770
O	5.014574	0.142808	-0.662027
C	3.635202	-1.720793	-0.307278
O	4.807068	-2.373614	-0.234535
C	4.832643	-3.784187	-0.020080
C	2.428362	-2.364531	-0.228462
C	1.105574	-1.872072	-0.288486
C	0.597994	-0.598475	-0.376508
C	-0.893129	-0.478723	-0.436229
C	-1.506250	0.214132	-1.490416
C	-2.894851	0.245808	-1.598570
C	-3.702408	-0.396221	-0.663735
O	-5.060121	-0.453349	-0.727932
C	-5.715499	0.183806	-1.821197

C	-3.109074	-1.047369	0.432018
O	-3.864586	-1.718723	1.356957
C	-4.681703	-0.910767	2.216144
C	-1.715511	-1.089739	0.530531
O	-1.162246	-1.789551	1.575855
C	-0.805384	-0.995061	2.714648
C	-0.648265	0.946925	-2.493332
C	0.035426	2.182341	-1.877235
C	2.199827	4.709331	1.581906
H	-0.213162	1.804500	0.226340
H	1.836027	3.645468	-0.713280
H	3.171514	1.681111	-0.531163
H	4.359613	-4.317239	-0.848616
H	5.885909	-4.048902	0.029690
H	4.341846	-4.048549	0.919881
H	2.471613	-3.441055	-0.121808
H	0.361644	-2.658553	-0.247708
H	-3.338871	0.766318	-2.436422
H	-6.777829	0.003536	-1.673359
H	-5.525462	1.260555	-1.824596
H	-5.402291	-0.247825	-2.775557
H	-5.448196	-0.381506	1.649461
H	-4.067751	-0.193246	2.768005
H	-5.150567	-1.599466	2.916871
H	-0.329992	-1.672028	3.422358
H	-0.106899	-0.200541	2.440108
H	-1.694376	-0.554421	3.173398
H	0.117250	0.270266	-2.883189
H	-1.251971	1.261329	-3.346354
H	0.837469	2.515767	-2.544833
H	-0.680022	3.004759	-1.790312

H	1.558769	5.406846	2.122731
H	3.031334	4.447501	2.238644
H	2.592900	5.199729	0.690684

Compound: Colchicine, Conformer: 04, Energy:-1359.466735 Hartree, Solvent: chloroform, Boltzmann %: 7.878

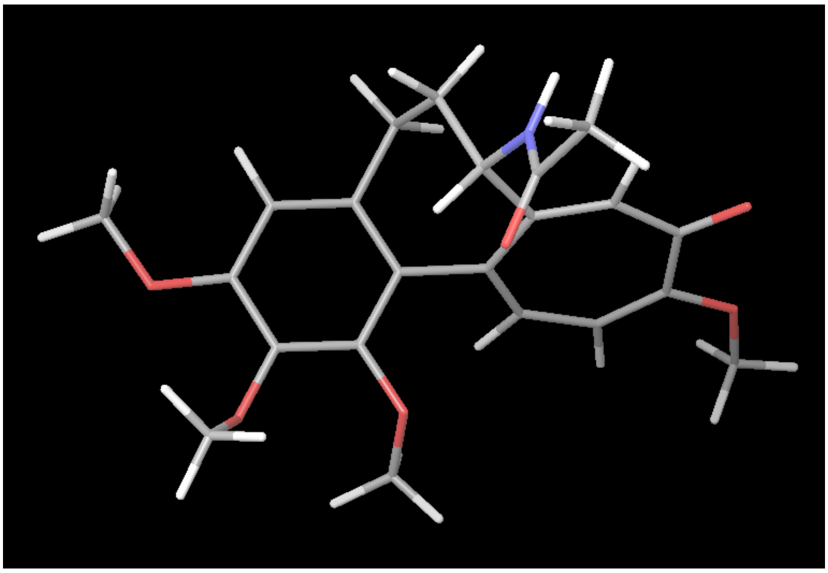

O	0.618221	2.983630	2.242722
C	1.253189	3.576324	1.377489
N	1.314158	3.143182	0.088678
C	0.607228	1.967108	-0.386135
C	1.417448	0.658154	-0.347474
C	2.778818	0.767847	-0.311567
C	3.893789	-0.155278	-0.298472
O	5.047744	0.297000	-0.296553
C	3.697445	-1.619489	-0.284637
O	4.880707	-2.255168	-0.241821
C	4.930552	-3.680481	-0.206434
C	2.502954	-2.288663	-0.322743
C	1.172591	-1.815086	-0.373761
C	0.636938	-0.551130	-0.379741
C	-0.855819	-0.455864	-0.456200
C	-1.462284	0.264337	-1.494937
C	-2.849424	0.315731	-1.608145
C	-3.661508	-0.317277	-0.670827
O	-5.020910	-0.311533	-0.694773

C	-5.675151	0.389000	-1.749121
C	-3.073120	-0.999776	0.405793
O	-3.866804	-1.596495	1.355949
C	-4.366263	-0.697512	2.358778
C	-1.680851	-1.088746	0.495658
O	-1.088428	-1.697524	1.571170
C	-1.370986	-3.086820	1.795594
C	-0.591270	1.023393	-2.465120
C	0.061896	2.250298	-1.801475
C	2.022332	4.844611	1.686064
H	-0.233761	1.835656	0.294896
H	1.846705	3.687709	-0.576523
H	3.165206	1.780401	-0.276777
H	4.492591	-4.112976	-1.109638
H	5.987378	-3.930915	-0.157868
H	4.419612	-4.070598	0.677411
H	2.565418	-3.369504	-0.324881
H	0.446288	-2.617427	-0.427623
H	-3.286081	0.861804	-2.433351
H	-6.740480	0.260292	-1.571829
H	-5.428451	1.454104	-1.730404
H	-5.415296	-0.031023	-2.724558
H	-4.995641	0.072548	1.908200
H	-3.540102	-0.233846	2.903857
H	-4.961763	-1.302735	3.040509
H	-2.402845	-3.237793	2.107296
H	-1.174612	-3.669769	0.890879
H	-0.688348	-3.401708	2.583051
H	0.189735	0.363892	-2.853039
H	-1.179270	1.352673	-3.323553
H	0.878173	2.608120	-2.438591

H	-0.665205	3.062541	-1.717303
H	1.340148	5.567270	2.135894
H	2.792709	4.613766	2.423969
H	2.493518	5.291886	0.810145

Compound: Colchicine, Conformer: 05, Energy:-1359.464055 Hartree, Solvent: chloroform, Boltzmann %: 0.461

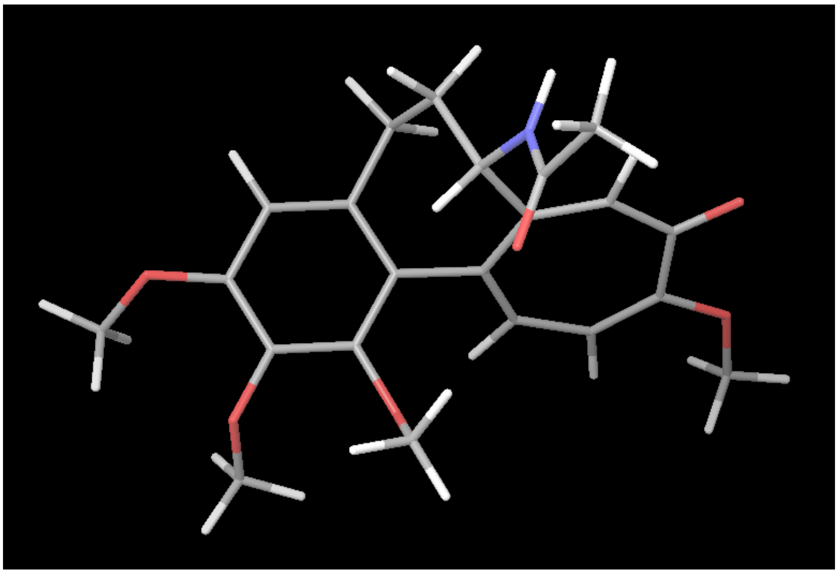

O	1.161648	2.813773	2.197899
C	1.809631	3.385265	1.327471
N	1.745461	3.023501	0.017472
C	0.872221	1.965932	-0.460187
C	1.480983	0.552174	-0.420921
C	2.843835	0.459793	-0.429436
C	3.807026	-0.620578	-0.431659
O	5.012807	-0.351474	-0.526896
C	3.395567	-2.034292	-0.297486
O	4.471819	-2.835303	-0.228432
C	4.311201	-4.244538	-0.072436
C	2.114520	-2.518292	-0.262539
C	0.868012	-1.856778	-0.332296
C	0.529651	-0.527572	-0.404338
C	-0.932257	-0.217784	-0.493662
C	-1.428147	0.579297	-1.543446
C	-2.790184	0.814479	-1.647270
C	-3.700369	0.280376	-0.736004
O	-5.008199	0.637866	-0.907784

C	-6.033435	-0.355597	-0.780240
C	-3.225407	-0.499018	0.326808
O	-4.093561	-0.926895	1.301876
C	-4.269573	-2.348011	1.424319
C	-1.843613	-0.727082	0.442535
O	-1.396093	-1.468701	1.513020
C	-1.206662	-0.709168	2.720067
C	-0.461580	1.200695	-2.520940
C	0.368450	2.323967	-1.872004
C	2.730793	4.542085	1.653582
H	0.020884	1.958506	0.220641
H	2.300979	3.535831	-0.654401
H	3.376097	1.404775	-0.439151
H	3.789315	-4.678744	-0.928912
H	5.320337	-4.645393	-0.018963
H	3.772634	-4.479938	0.848902
H	2.016165	-3.593847	-0.187748
H	0.028313	-2.541542	-0.323803
H	-3.182456	1.421687	-2.455280
H	-6.904259	0.055075	-1.288946
H	-5.733672	-1.283586	-1.274214
H	-6.271529	-0.550344	0.263915
H	-3.329607	-2.838967	1.673201
H	-4.672417	-2.764715	0.496896
H	-4.988689	-2.492085	2.228933
H	-0.856907	-1.415447	3.470995
H	-0.455266	0.070206	2.570467
H	-2.147835	-0.260012	3.044135
H	0.212963	0.432120	-2.908434
H	-1.002666	1.605547	-3.377634
H	1.224062	2.553893	-2.516352

H	-0.232038	3.233929	-1.788687
H	2.153447	5.316658	2.160489
H	3.496982	4.193314	2.348058
H	3.215611	4.972325	0.776616

Compound: Colchicine, Conformer: 06, Energy:-1359.466024 Hartree, Solvent: chloroform, Boltzmann %: 3.71

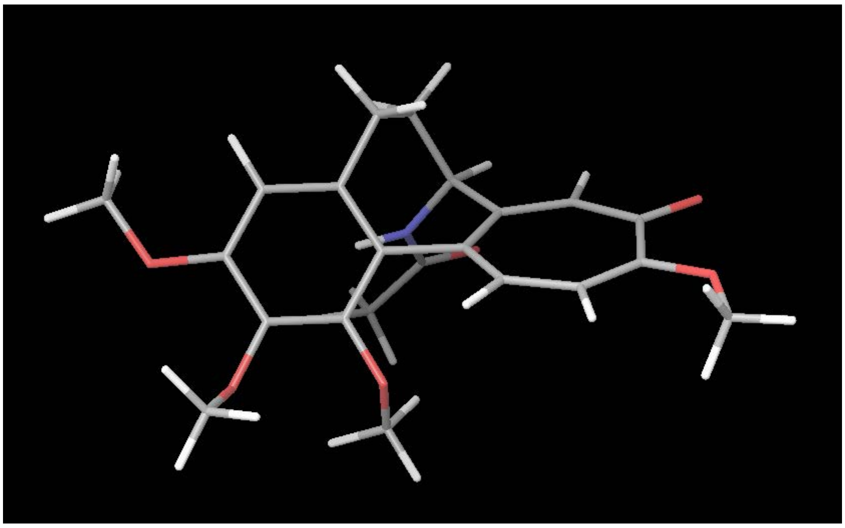

O	1.053059	-3.846944	1.701467
C	0.006526	-3.219635	1.570518
N	-0.232798	-2.388292	0.521048
C	0.720584	-2.210538	-0.574443
C	1.549876	-0.919988	-0.455107
C	2.903199	-1.130508	-0.440127
C	4.084572	-0.292559	-0.383281
O	5.200594	-0.825473	-0.439283
C	3.995961	1.176271	-0.244303
O	5.221262	1.718048	-0.152937
C	5.375114	3.128331	0.000660
C	2.853188	1.930815	-0.224435
C	1.491916	1.562006	-0.310383
C	0.862793	0.343819	-0.405814
C	-0.631634	0.382346	-0.490348
C	-1.306220	-0.254567	-1.544159
C	-2.695344	-0.183202	-1.644110
C	-3.447737	0.514440	-0.702167
O	-4.800273	0.636227	-0.724494
C	-5.517684	0.018132	-1.790380

C	-2.791025	1.151729	0.362496
O	-3.513190	1.789750	1.339018
C	-3.879065	3.141439	1.024840
C	-1.399477	1.086969	0.458814
O	-0.785389	1.764587	1.484377
C	-0.827415	1.111333	2.761748
C	-0.517117	-1.051594	-2.552283
C	0.053539	-2.354290	-1.962564
C	-1.111635	-3.333517	2.587376
H	1.410550	-3.042629	-0.456112
H	-1.125254	-1.919981	0.475072
H	3.214388	-2.170304	-0.481184
H	4.889188	3.480138	0.914134
H	6.447165	3.295378	0.068895
H	4.974631	3.664481	-0.863371
H	2.993537	3.001259	-0.142841
H	0.827847	2.417575	-0.299518
H	-3.181379	-0.679063	-2.473620
H	-6.565847	0.245167	-1.610196
H	-5.217014	0.426309	-2.759053
H	-5.374302	-1.065705	-1.787604
H	-4.502779	3.174788	0.128865
H	-2.986598	3.756443	0.883815
H	-4.445982	3.510137	1.878062
H	-0.278424	0.166428	2.725437
H	-0.339091	1.785338	3.463324
H	-1.858330	0.937831	3.074513
H	-1.144372	-1.294203	-3.412122
H	0.308235	-0.441033	-2.929752
H	-0.734098	-3.107718	-1.875356
H	0.797576	-2.752965	-2.657043

H	-0.725114	-3.045338	3.566280
H	-1.980877	-2.718891	2.350799
H	-1.420060	-4.378363	2.650774

Compound: Colchicine, Conformer: 07, Energy:-1359.465020 Hartree, Solvent: chloroform, Boltzmann %: 1.281

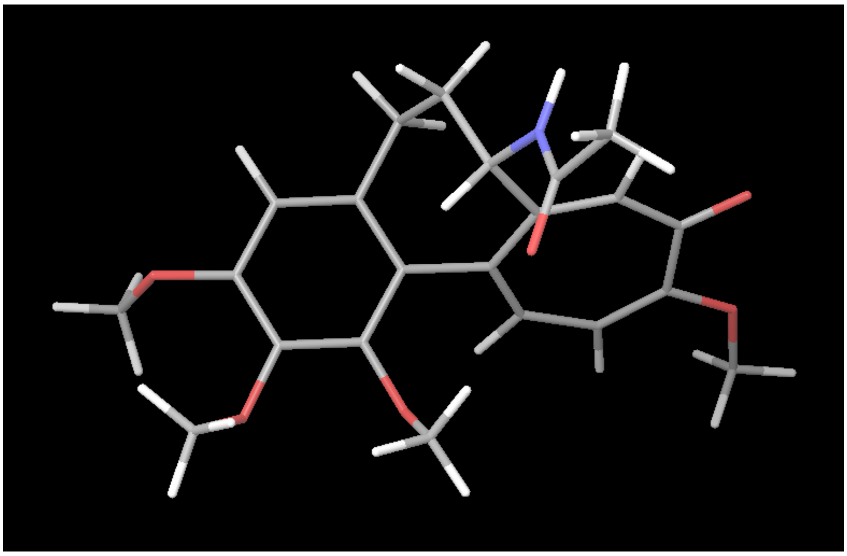

O	1.036958	2.888676	2.005454
C	1.635227	3.459412	1.099323
N	1.566515	3.039525	-0.192983
C	0.760475	1.902268	-0.599526
C	1.453843	0.534675	-0.463908
C	2.819431	0.522802	-0.483723
C	3.843417	-0.497712	-0.419568
O	5.029849	-0.170209	-0.561369
C	3.517040	-1.916377	-0.158836
O	4.639494	-2.641909	-0.027281
C	4.564692	-4.038648	0.255671
C	2.267289	-2.471650	-0.076747
C	0.983262	-1.892739	-0.191193
C	0.568850	-0.594357	-0.360172
C	-0.909340	-0.374939	-0.466600
C	-1.443243	0.298900	-1.580704
C	-2.820646	0.418416	-1.718916
C	-3.689493	-0.097438	-0.765474
O	-5.044830	0.049084	-0.954477
C	-5.747054	-1.167884	-1.255177

C	-3.177524	-0.705409	0.386937
O	-3.992150	-1.218362	1.363059
C	-4.791654	-0.265196	2.082306
C	-1.788482	-0.858163	0.517490
O	-1.305030	-1.529783	1.613982
C	-1.009454	-0.705201	2.749976
C	-0.509943	0.907440	-2.598405
C	0.241462	2.127873	-2.032673
C	2.497456	4.676535	1.358993
H	-0.091974	1.888846	0.079669
H	2.084473	3.547137	-0.897624
H	3.294741	1.493767	-0.571974
H	4.072044	-4.579102	-0.556463
H	5.596292	-4.370768	0.341554
H	4.039340	-4.222126	1.196199
H	2.233404	-3.541578	0.085249
H	0.185276	-2.622561	-0.124615
H	-3.246092	0.909038	-2.587442
H	-6.795139	-0.894367	-1.362935
H	-5.381596	-1.593354	-2.193933
H	-5.634455	-1.896733	-0.450547
H	-5.501076	0.236167	1.423305
H	-4.154390	0.479105	2.567617
H	-5.325157	-0.835625	2.840388
H	-0.288938	0.075227	2.491717
H	-1.919586	-0.247061	3.144568
H	-0.580957	-1.365095	3.502299
H	0.216267	0.158142	-2.925594
H	-1.069150	1.211274	-3.484758
H	1.083035	2.365981	-2.692287
H	-0.417556	3.000273	-2.015073

H	1.896123	5.429535	1.870208
H	3.310214	4.391547	2.029460
H	2.921276	5.110033	0.452500

Compound: Colchicine, Conformer: 08, Energy:-1359.465431 Hartree, Solvent: chloroform, Boltzmann %: 1.98

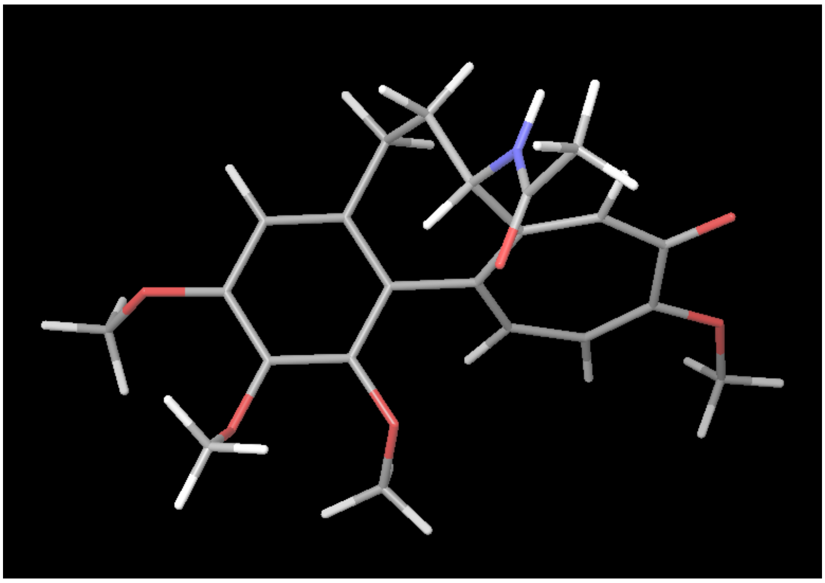

O	0.890182	2.970736	2.146878
C	1.544828	3.530383	1.274387
N	1.561183	3.097912	-0.016117
C	0.772328	1.969576	-0.477816
C	1.473942	0.602056	-0.387872
C	2.839461	0.603093	-0.350689
C	3.874785	-0.407406	-0.301374
O	5.061826	-0.054774	-0.342648
C	3.557965	-1.846777	-0.187410
O	4.683724	-2.571280	-0.077212
C	4.615770	-3.988693	0.070404
C	2.313571	-2.418505	-0.208455
C	1.027637	-1.842072	-0.313683
C	0.598577	-0.540614	-0.386334
C	-0.880442	-0.327186	-0.499727
C	-1.402468	0.407082	-1.579881
C	-2.776366	0.559914	-1.719528
C	-3.652844	0.033967	-0.778403
O	-4.997249	0.281256	-0.913251

C	-5.815687	-0.869643	-1.174644
C	-3.150986	-0.645347	0.335808
O	-4.007249	-1.101216	1.311612
C	-4.343873	-0.111214	2.299207
C	-1.768266	-0.852888	0.456844
O	-1.250335	-1.460894	1.570386
C	-1.657729	-2.809983	1.844602
C	-0.452686	1.060027	-2.552705
C	0.281165	2.253443	-1.912183
C	2.386991	4.752837	1.575053
H	-0.090807	1.923169	0.186540
H	2.115650	3.609350	-0.689553
H	3.305910	1.582128	-0.355617
H	4.162719	-4.455265	-0.808025
H	5.647269	-4.318309	0.166675
H	4.056263	-4.264562	0.967750
H	2.287355	-3.499088	-0.147278
H	0.239348	-2.584810	-0.346411
H	-3.188653	1.107196	-2.559775
H	-6.837864	-0.502330	-1.246611
H	-5.526511	-1.331252	-2.123195
H	-5.739501	-1.597634	-0.366267
H	-4.845397	0.740090	1.833908
H	-3.447327	0.225580	2.825122
H	-5.020015	-0.597976	3.000140
H	-2.709068	-2.859666	2.122608
H	-1.479025	-3.449336	0.975055
H	-1.033282	-3.142890	2.671791
H	0.281681	0.328532	-2.900669
H	-0.995609	1.405283	-3.434011
H	1.136061	2.525713	-2.540809

H	-0.382012	3.121637	-1.868918
H	1.747987	5.517010	2.019872
H	3.142664	4.482468	2.314582
H	2.882661	5.166669	0.696194

S8. Optimized coordinates for colchicine in DMSO solvent (B3LYP/6311+G(2d,p))

Compound: Colchicine, Conformer: 01, Energy:-1359.477937 Hartree, Solvent: dmso, Boltzmann %: 62.714

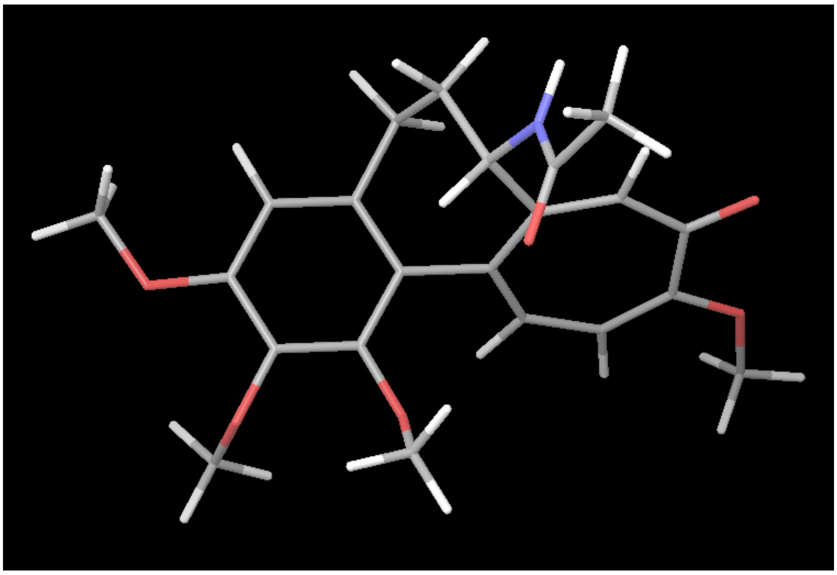

O	1.122630	2.792799	2.284494
C	1.681848	3.417700	1.384502
N	1.566837	3.070595	0.078185
C	0.737684	1.967577	-0.376929
C	1.432740	0.593388	-0.389898
C	2.799709	0.588283	-0.440336
C	3.827484	-0.425684	-0.484759
O	5.016810	-0.073202	-0.577695
C	3.512822	-1.864532	-0.405338
O	4.639407	-2.599103	-0.403649
C	4.569737	-4.024229	-0.320343
C	2.266159	-2.431699	-0.353596
C	0.979429	-1.851197	-0.356211
C	0.552914	-0.544025	-0.370062
C	-0.927912	-0.327588	-0.392738
C	-1.516075	0.455281	-1.399291
C	-2.898562	0.614153	-1.458450

C	-3.729625	0.004967	-0.520469
O	-5.084932	0.100783	-0.507502
C	-5.717668	0.867844	-1.530968
C	-3.158593	-0.771097	0.499515
O	-3.955381	-1.327759	1.470453
C	-4.470543	-2.626804	1.141415
C	-1.772871	-0.931756	0.557599
O	-1.246000	-1.729497	1.545509
C	-1.168756	-1.116724	2.842278
C	-0.630052	1.157692	-2.398920
C	0.153088	2.318516	-1.759502
C	2.541498	4.628010	1.680084
H	-0.082055	1.895194	0.338416
H	2.053190	3.626936	-0.615869
H	3.267847	1.566960	-0.447699
H	4.045387	-4.442848	-1.182611
H	5.602574	-4.363524	-0.320433
H	4.078441	-4.340080	0.602997
H	2.237479	-3.513723	-0.321565
H	0.187368	-2.590526	-0.346342
H	-3.317592	1.219629	-2.251218
H	-6.784569	0.802739	-1.330324
H	-5.402495	1.913816	-1.492931
H	-5.506348	0.455880	-2.521219
H	-3.653357	-3.336960	0.993161
H	-5.092006	-2.579165	0.244146
H	-5.077532	-2.939528	1.989580
H	-0.741414	-1.865681	3.506817
H	-0.515770	-0.240151	2.811641
H	-2.161012	-0.832227	3.195645
H	0.075019	0.440898	-2.829042

H	-1.228410	1.544397	-3.225365
H	0.965110	2.615587	-2.431766
H	-0.498945	3.186867	-1.632003
H	1.937065	5.365815	2.210315
H	3.353752	4.327375	2.344085
H	2.963406	5.087525	0.785916

Compound: Colchicine, Conformer: 02, Energy:-1359.475928 Hartree, Solvent: dmso, Boltzmann %: 7.47

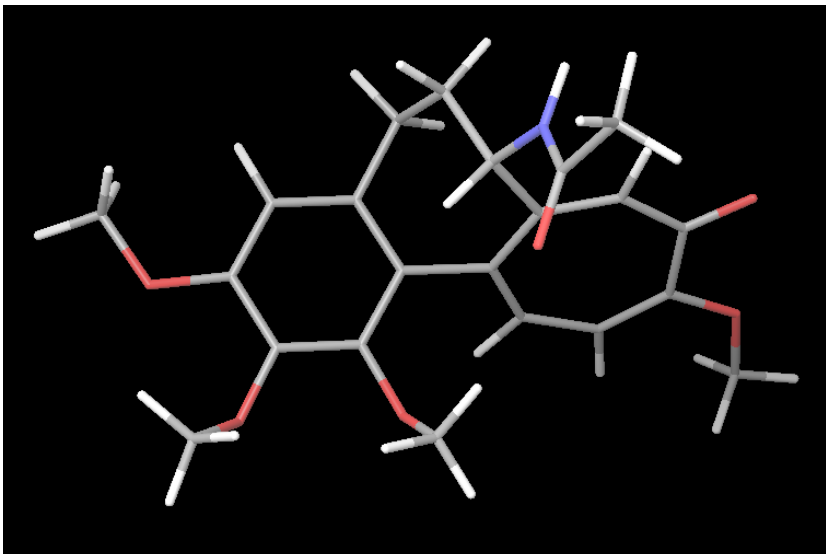

O	0.929177	2.808834	2.174527
C	1.450305	3.457352	1.268476
N	1.365930	3.082913	-0.032823
C	0.617169	1.916851	-0.468771
C	1.398224	0.590949	-0.427978
C	2.763078	0.668893	-0.472084
C	3.849879	-0.282626	-0.474870
O	5.015452	0.134177	-0.595619
C	3.622679	-1.731529	-0.313029
O	4.791920	-2.392336	-0.250704
C	4.809148	-3.810951	-0.076312
C	2.412793	-2.372684	-0.251405
C	1.093227	-1.874195	-0.302574
C	0.589765	-0.596728	-0.374909
C	-0.901338	-0.473440	-0.431266
C	-1.513764	0.224976	-1.482433
C	-2.902230	0.260189	-1.591090
C	-3.710762	-0.385815	-0.659957
O	-5.067903	-0.440080	-0.722925
C	-5.725400	0.228278	-1.798820

C	-3.117838	-1.046423	0.430678
O	-3.879606	-1.723176	1.349027
C	-4.672032	-0.911357	2.230319
C	-1.724806	-1.091291	0.530409
O	-1.175110	-1.803171	1.571150
C	-0.786632	-1.018158	2.706226
C	-0.654821	0.955267	-2.486023
C	0.035477	2.186236	-1.870508
C	2.224537	4.727385	1.549277
H	-0.209166	1.812559	0.234937
H	1.819275	3.657135	-0.734580
H	3.170291	1.673680	-0.515999
H	4.333345	-4.316089	-0.920166
H	5.860819	-4.082952	-0.033317
H	4.315844	-4.097882	0.855339
H	2.450473	-3.451511	-0.166522
H	0.346809	-2.659196	-0.273898
H	-3.343851	0.786538	-2.426905
H	-6.788267	0.055648	-1.646397
H	-5.522896	1.302222	-1.779133
H	-5.422952	-0.185709	-2.764122
H	-5.414870	-0.337851	1.675385
H	-4.034580	-0.234369	2.805668
H	-5.170372	-1.602538	2.907857
H	-0.322230	-1.706734	3.410049
H	-0.069668	-0.243260	2.423454
H	-1.658908	-0.552949	3.172708
H	0.106126	0.275106	-2.879055
H	-1.259606	1.274129	-3.336413
H	0.837616	2.517365	-2.538703
H	-0.676072	3.011588	-1.781058

H	1.569440	5.429693	2.067385
H	3.052956	4.492009	2.219654
H	2.617544	5.201930	0.649802

Compound: Colchicine, Conformer: 03, Energy:-1359.475922 Hartree, Solvent: dmso, Boltzmann %: 7.422

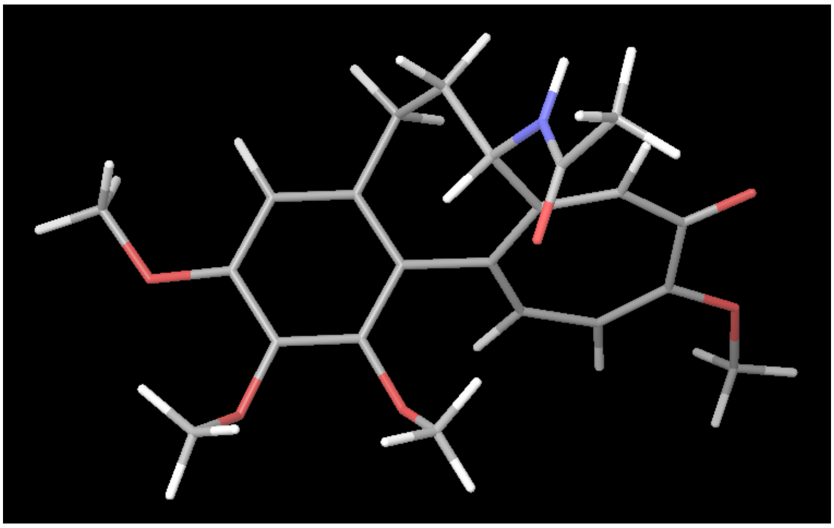

O	0.928195	2.808997	2.174414
C	1.449440	3.457541	1.268451
N	1.365485	3.082979	-0.032848
C	0.617009	1.916763	-0.468875
C	1.398264	0.590981	-0.427903
C	2.763118	0.669119	-0.471782
C	3.850044	-0.282258	-0.474594
O	5.015568	0.134710	-0.595279
C	3.623040	-1.731208	-0.312872
O	4.792368	-2.391865	-0.250682
C	4.809801	-3.810459	-0.076131
C	2.413234	-2.372522	-0.251163
C	1.093605	-1.874212	-0.302389
C	0.589971	-0.596815	-0.374887
C	-0.901135	-0.473712	-0.431406
C	-1.513573	0.224628	-1.482636
C	-2.902028	0.259809	-1.591269
C	-3.710569	-0.386128	-0.660069
O	-5.067716	-0.440495	-0.723112
C	-5.725277	0.228277	-1.798717

C	-3.117644	-1.046659	0.430600
O	-3.879272	-1.723412	1.349046
C	-4.672249	-0.911718	2.229979
C	-1.724588	-1.091519	0.530283
O	-1.174789	-1.803318	1.571042
C	-0.787240	-1.018157	2.706339
C	-0.654609	0.954900	-2.486222
C	0.035514	2.185984	-1.870729
C	2.223217	4.727834	1.549356
H	-0.209438	1.812387	0.234687
H	1.818944	3.657195	-0.734531
H	3.170203	1.673962	-0.515577
H	4.334066	-4.315760	-0.919931
H	5.861514	-4.082302	-0.033109
H	4.316538	-4.097355	0.855553
H	2.451065	-3.451341	-0.166235
H	0.347284	-2.659303	-0.273686
H	-3.343678	0.786060	-2.427131
H	-6.788157	0.055888	-1.646098
H	-5.522484	1.302164	-1.778835
H	-5.423157	-0.185577	-2.764182
H	-5.415631	-0.339143	1.674819
H	-4.035265	-0.233872	2.804837
H	-5.169904	-1.602928	2.907992
H	-0.323237	-1.706601	3.410556
H	-0.070176	-0.243187	2.423967
H	-1.659899	-0.553004	3.172156
H	0.106440	0.274772	-2.879116
H	-1.259339	1.273634	-3.336704
H	0.837731	2.517114	-2.538834
H	-0.676112	3.011292	-1.781455

H	1.567280	5.430549	2.065873
H	3.050600	4.493072	2.221214
H	2.617539	5.201689	0.650090

Compound: Colchicine, Conformer: 04, Energy:-1359.476643 Hartree, Solvent: dmso, Boltzmann %: 15.928

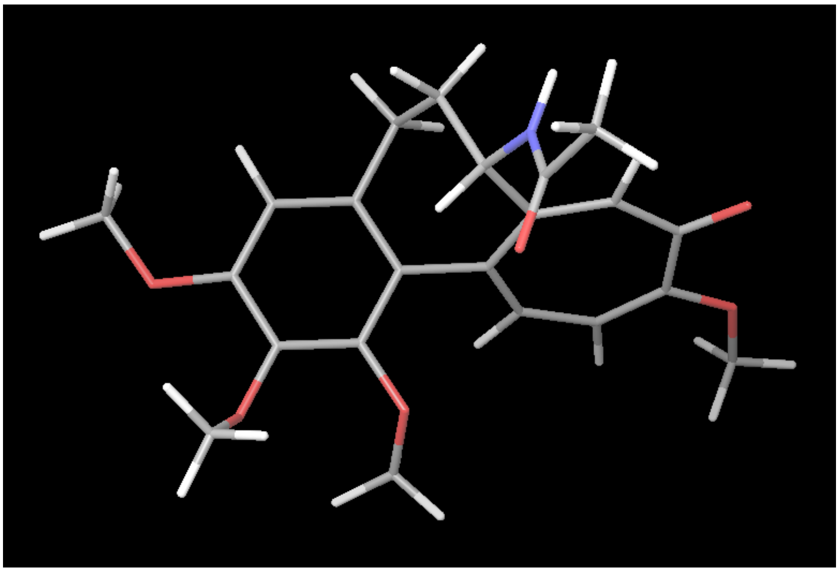

O	0.806024	2.853722	2.276879
C	1.354627	3.511545	1.394088
N	1.326889	3.142238	0.089054
C	0.608772	1.972455	-0.387052
C	1.410160	0.658184	-0.355940
C	2.774079	0.759024	-0.330343
C	3.878313	-0.172326	-0.314299
O	5.040080	0.271959	-0.306405
C	3.672824	-1.632791	-0.304526
O	4.851615	-2.278512	-0.266780
C	4.890094	-3.706982	-0.243157
C	2.473403	-2.294889	-0.340939
C	1.147409	-1.812766	-0.384359
C	0.621088	-0.543482	-0.383002
C	-0.871117	-0.436902	-0.453075
C	-1.476036	0.287032	-1.490692
C	-2.863010	0.342403	-1.602638
C	-3.676061	-0.290015	-0.665511
O	-5.035006	-0.284223	-0.690867

C	-5.688821	0.427506	-1.740778
C	-3.088553	-0.972881	0.411869
O	-3.884369	-1.567670	1.362658
C	-4.375412	-0.668088	2.369873
C	-1.696595	-1.064890	0.501153
O	-1.103855	-1.671478	1.579444
C	-1.388738	-3.061831	1.807812
C	-0.603999	1.043626	-2.462160
C	0.059780	2.264924	-1.798679
C	2.102577	4.789270	1.710041
H	-0.232789	1.840299	0.293586
H	1.799118	3.727690	-0.590623
H	3.164448	1.770964	-0.306780
H	4.444991	-4.126921	-1.148297
H	5.945054	-3.966004	-0.200475
H	4.378756	-4.098948	0.639214
H	2.527176	-3.376322	-0.348024
H	0.416212	-2.610812	-0.440551
H	-3.297853	0.890817	-2.427608
H	-6.754222	0.301357	-1.562543
H	-5.437065	1.490880	-1.713533
H	-5.430945	0.013454	-2.718961
H	-4.994582	0.111908	1.922378
H	-3.544678	-0.217118	2.918678
H	-4.978604	-1.269548	3.048115
H	-2.414981	-3.206531	2.139862
H	-1.212387	-3.643294	0.898660
H	-0.692507	-3.381395	2.581293
H	0.170956	0.380233	-2.855756
H	-1.194018	1.379592	-3.316340
H	0.875673	2.619529	-2.437559

H	-0.661996	3.081212	-1.708775
H	1.419919	5.481143	2.206130
H	2.907523	4.560065	2.410491
H	2.523970	5.272951	0.828488

Compound: Colchicine, Conformer: 15, Energy:-1359.473101 Hartree, Solvent: dmso, Boltzmann %: 0.374

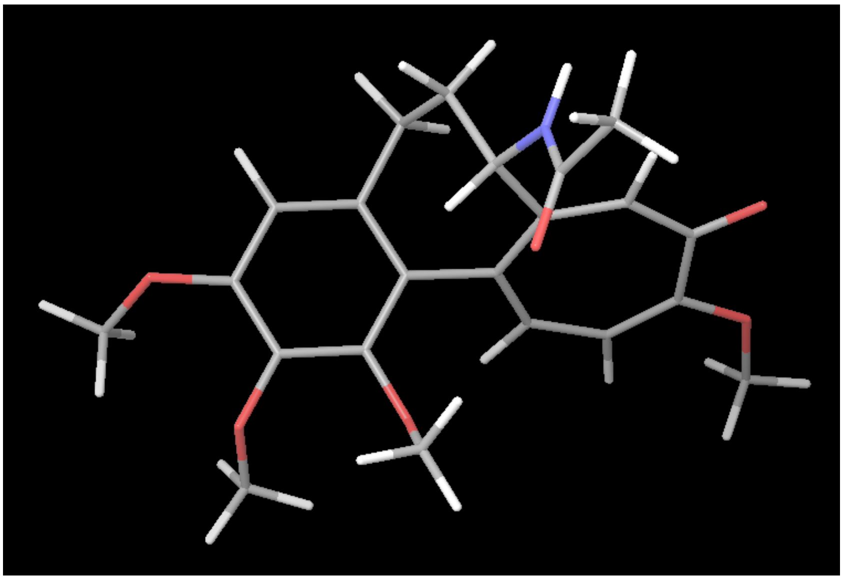

O	1.229976	2.811637	2.190917
C	1.860696	3.384602	1.303745
N	1.773446	3.019719	-0.000064
C	0.886562	1.966694	-0.464137
C	1.481845	0.547415	-0.417738
C	2.845682	0.442670	-0.418530
C	3.796822	-0.644510	-0.400332
O	5.011311	-0.382798	-0.456493
C	3.374114	-2.055072	-0.300160
O	4.442279	-2.869016	-0.241530
C	4.265846	-4.283174	-0.129860
C	2.088398	-2.529272	-0.279627
C	0.848993	-1.856731	-0.339037
C	0.522302	-0.522840	-0.399656
C	-0.937956	-0.202766	-0.481837
C	-1.432598	0.595604	-1.530790
C	-2.794489	0.838562	-1.629336
C	-3.701747	0.307244	-0.714701
O	-5.013293	0.666511	-0.870083

C	-6.018572	-0.357830	-0.841239
C	-3.228214	-0.476975	0.344981
O	-4.099898	-0.907691	1.315277
C	-4.277491	-2.331187	1.430002
C	-1.847380	-0.710012	0.457945
O	-1.396422	-1.456634	1.523652
C	-1.237290	-0.713521	2.744281
C	-0.466184	1.205765	-2.515349
C	0.379311	2.322252	-1.875683
C	2.780598	4.546409	1.611943
H	0.037553	1.970394	0.220111
H	2.316238	3.532383	-0.685835
H	3.383767	1.384721	-0.432795
H	3.741393	-4.683173	-1.000815
H	5.270615	-4.695938	-0.086477
H	3.722347	-4.540106	0.782392
H	1.979343	-3.605078	-0.223442
H	0.003675	-2.535116	-0.337187
H	-3.185560	1.447385	-2.437206
H	-6.897311	0.077878	-1.314008
H	-5.694624	-1.228852	-1.416577
H	-6.255731	-0.652296	0.179658
H	-3.340921	-2.822232	1.690663
H	-4.665913	-2.743585	0.494977
H	-5.008344	-2.477904	2.223317
H	-0.884495	-1.424627	3.489195
H	-0.497511	0.080863	2.615281
H	-2.189739	-0.285513	3.063667
H	0.197570	0.429148	-2.905716
H	-1.008840	1.614351	-3.369190
H	1.234214	2.540873	-2.524403

H	-0.210568	3.239133	-1.793018
H	2.199905	5.333537	2.095920
H	3.541087	4.213669	2.320363
H	3.270626	4.956029	0.728334

Compound: Colchicine, Conformer: 06, Energy:-1359.473789 Hartree, Solvent: dmso, Boltzmann %: 0.775

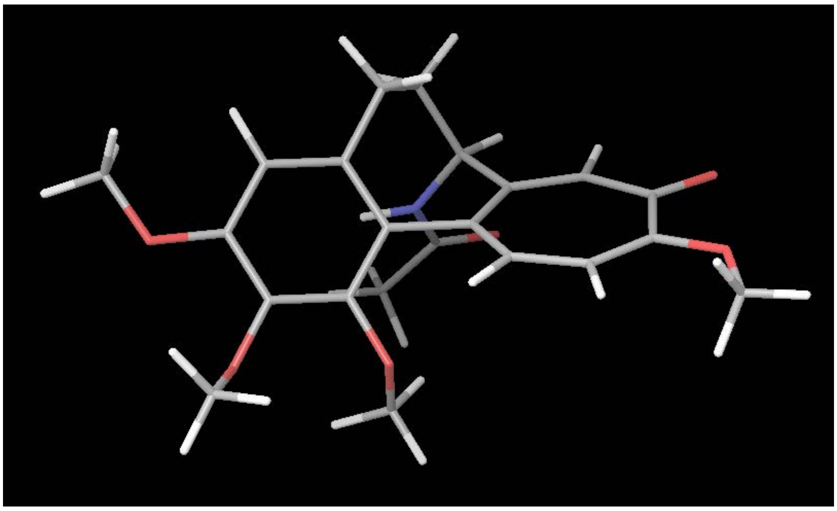

O	1.232366	-3.679561	1.745185
C	0.116930	-3.186671	1.577847
N	-0.199533	-2.434557	0.494612
C	0.735386	-2.219682	-0.611071
C	1.543604	-0.916580	-0.477582
C	2.901800	-1.106961	-0.474941
C	4.069125	-0.257402	-0.399265
O	5.196100	-0.780668	-0.445063
C	3.963931	1.206216	-0.258571
O	5.182973	1.766786	-0.178598
C	5.317071	3.182636	-0.034392
C	2.810371	1.945383	-0.222557
C	1.454668	1.559902	-0.296055
C	0.839240	0.333516	-0.400473
C	-0.655350	0.357022	-0.476583
C	-1.327179	-0.265857	-1.540432
C	-2.715376	-0.191017	-1.645123
C	-3.469580	0.497066	-0.697515
O	-4.821351	0.625122	-0.726039
C	-5.534203	0.027047	-1.808352
C	-2.815474	1.117533	0.378218

O	-3.545041	1.750298	1.354644
C	-3.811073	3.136673	1.090347
C	-1.425159	1.046733	0.480962
O	-0.813359	1.706966	1.520314
C	-0.871893	1.037294	2.788926
C	-0.536028	-1.045579	-2.560206
C	0.045986	-2.350824	-1.989152
C	-0.992894	-3.398161	2.586381
H	1.445390	-3.039039	-0.523544
H	-1.143876	-2.080553	0.415142
H	3.223826	-2.142526	-0.539686
H	4.838826	3.530619	0.884186
H	6.387264	3.366558	0.017185
H	4.895064	3.706389	-0.895326
H	2.936113	3.017480	-0.136249
H	0.780342	2.407438	-0.271382
H	-3.198049	-0.675866	-2.483373
H	-6.582471	0.257268	-1.632843
H	-5.222862	0.449206	-2.767357
H	-5.395788	-1.057147	-1.821283
H	-4.391468	3.248880	0.171556
H	-2.876933	3.698214	1.013926
H	-4.390953	3.503734	1.935614
H	-0.329991	0.088740	2.744619
H	-0.385508	1.698545	3.503940
H	-1.906480	0.865805	3.089870
H	-1.165698	-1.284332	-3.419177
H	0.283248	-0.425192	-2.935468
H	-0.738760	-3.106912	-1.897658
H	0.780145	-2.742150	-2.697998
H	-0.651820	-3.052169	3.563452

H	-1.918592	-2.883829	2.326900
H	-1.191145	-4.468528	2.666678

Compound: Colchicine, Conformer: 07, Energy:-1359.474317 Hartree, Solvent: dmso, Boltzmann %: 1.356

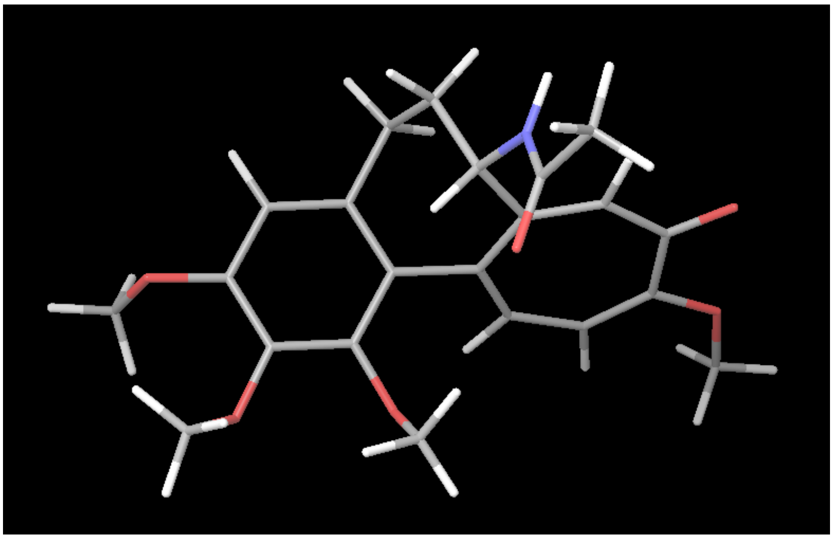

O	1.017339	2.973174	1.979567
C	1.622915	3.513797	1.055480
N	1.577762	3.042210	-0.215853
C	0.768369	1.899798	-0.602795
C	1.456924	0.531517	-0.445770
C	2.824372	0.520006	-0.425046
C	3.845335	-0.496239	-0.317916
O	5.040246	-0.155489	-0.361208
C	3.517013	-1.925700	-0.149741
O	4.637317	-2.659765	-0.039836
C	4.556890	-4.075562	0.140528
C	2.266103	-2.484639	-0.114072
C	0.984762	-1.900778	-0.215737
C	0.571691	-0.597684	-0.358750
C	-0.906998	-0.379211	-0.463415
C	-1.441345	0.297514	-1.575065
C	-2.818687	0.423813	-1.708459
C	-3.686564	-0.091419	-0.753733
O	-5.041993	0.065226	-0.935001
C	-5.751670	-1.142526	-1.258759

C	-3.173947	-0.712307	0.391687
O	-3.986948	-1.235242	1.364487
C	-4.787601	-0.289895	2.095026
C	-1.785123	-0.870655	0.517130
O	-1.301745	-1.561997	1.602839
C	-1.005142	-0.760608	2.754500
C	-0.507228	0.894910	-2.597964
C	0.254543	2.111735	-2.040890
C	2.463010	4.752729	1.278567
H	-0.086465	1.898651	0.074129
H	2.096183	3.533394	-0.935324
H	3.298556	1.492931	-0.499472
H	4.070124	-4.552915	-0.713161
H	5.586998	-4.415577	0.211578
H	4.022630	-4.324140	1.060504
H	2.228853	-3.560717	0.000504
H	0.186082	-2.632109	-0.173799
H	-3.243604	0.918251	-2.575530
H	-6.798280	-0.861358	-1.360252
H	-5.388727	-1.551814	-2.205422
H	-5.642843	-1.886397	-0.467533
H	-5.503162	0.211116	1.442858
H	-4.150745	0.454184	2.580960
H	-5.313977	-0.867635	2.852449
H	-0.275497	0.016386	2.511505
H	-1.912130	-0.299299	3.152326
H	-0.586019	-1.436631	3.497740
H	0.211463	0.137779	-2.924073
H	-1.066901	1.199346	-3.483724
H	1.100244	2.337229	-2.699185
H	-0.395967	2.990477	-2.032573

H	1.828267	5.535350	1.697522
H	3.235937	4.524750	2.014490
H	2.935173	5.124448	0.368839

Compound: Colchicine, Conformer: 08, Energy:-1359.475329 Hartree, Solvent: dmso, Boltzmann %: 3.961

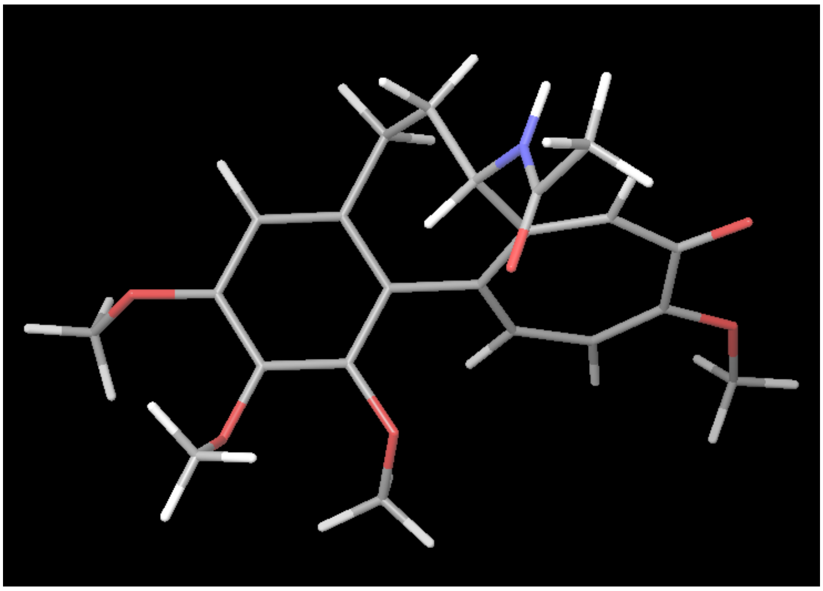

O	0.979808	2.937918	2.134844
C	1.595085	3.518686	1.242007
N	1.570786	3.099425	-0.048147
C	0.775962	1.968615	-0.495068
C	1.470909	0.599281	-0.384633
C	2.837308	0.595099	-0.327374
C	3.864079	-0.417769	-0.242651
O	5.055922	-0.064507	-0.218580
C	3.544593	-1.857452	-0.183858
O	4.667694	-2.590562	-0.094194
C	4.593871	-4.016146	-0.019617
C	2.297899	-2.425277	-0.222861
C	1.014980	-1.843166	-0.315310
C	0.591781	-0.538055	-0.379612
C	-0.886642	-0.318952	-0.490170
C	-1.407998	0.405444	-1.576969
C	-2.782119	0.559058	-1.715113
C	-3.656822	0.041152	-0.767874
O	-5.002850	0.282961	-0.903131

C	-5.811981	-0.872328	-1.180700
C	-3.154870	-0.628083	0.352870
O	-4.008763	-1.082789	1.330833
C	-4.398921	-0.078186	2.283084
C	-1.771910	-0.832767	0.474559
O	-1.249670	-1.426585	1.594954
C	-1.649787	-2.777350	1.882398
C	-0.457273	1.043161	-2.558913
C	0.287819	2.237633	-1.933448
C	2.425481	4.751658	1.527558
H	-0.090506	1.933246	0.166039
H	2.098441	3.621749	-0.738454
H	3.305338	1.573755	-0.337050
H	4.134447	-4.432979	-0.918972
H	5.624363	-4.354212	0.055092
H	4.036848	-4.334425	0.864712
H	2.266210	-3.506976	-0.185683
H	0.224252	-2.583392	-0.354602
H	-3.194542	1.097222	-2.561649
H	-6.837325	-0.513362	-1.248726
H	-5.517200	-1.318971	-2.134374
H	-5.729804	-1.609405	-0.381178
H	-4.920492	0.742010	1.785577
H	-3.524776	0.303841	2.816125
H	-5.070754	-0.568774	2.985367
H	-2.697516	-2.826273	2.173160
H	-1.478597	-3.420240	1.014633
H	-1.014918	-3.103477	2.704273
H	0.270074	0.302806	-2.903104
H	-1.001030	1.384570	-3.441069
H	1.145123	2.495088	-2.564475

H	-0.367453	3.112201	-1.900600
H	1.781385	5.512295	1.971917
H	3.191040	4.496469	2.262272
H	2.906096	5.164242	0.640160

S9. Optimized coordinates for the cutdown colchicine (monomer 2) in chloroform solvent (B3LYP/6311+G(2d,p))

Compound: monomer2, Conformer: 01, Energy:-1015.889989 Hartree, Solvent: chloroform, Boltzmann %: 49.069

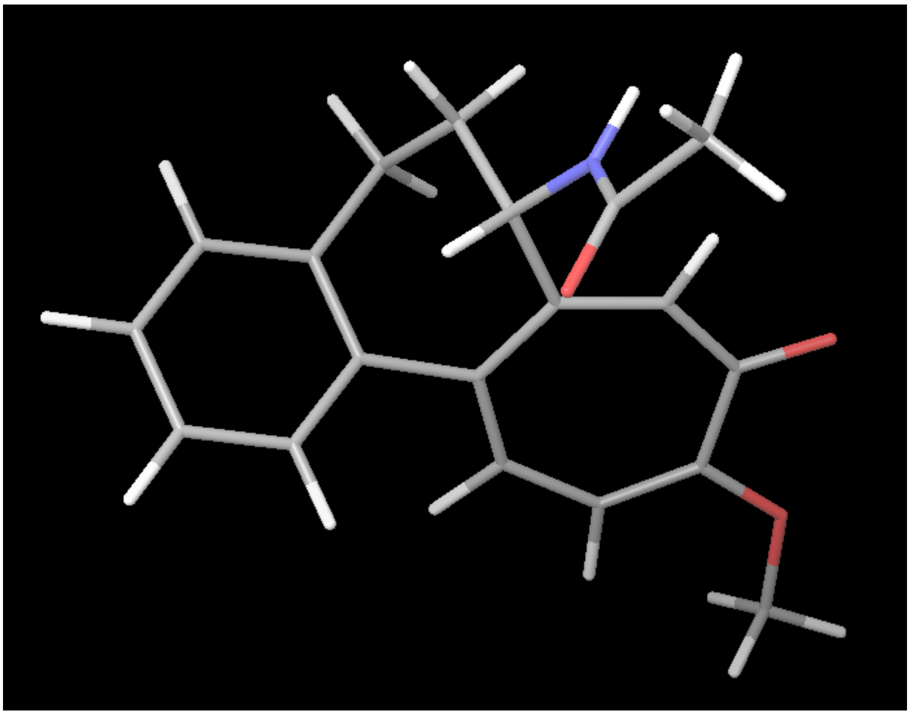

O	1.050251	2.750428	-1.981368
C	0.846593	3.347707	-0.929976
N	0.820242	2.714199	0.274164
C	1.073043	1.291476	0.419452
C	-0.173095	0.396433	0.284368
C	-1.386111	0.974562	0.522363
C	-2.762793	0.523120	0.529220
O	-3.645384	1.294524	0.927913
C	-3.148553	-0.813808	0.028752
O	-4.485697	-0.937347	-0.008068
C	-5.083008	-2.140950	-0.488457
C	-2.303629	-1.827900	-0.335469
C	-0.891461	-1.901446	-0.344178
C	0.086038	-0.974351	-0.076186
C	1.498914	-1.454346	-0.190109

C	2.419936	-1.241562	0.854449
C	3.710103	-1.755632	0.738910
C	4.106322	-2.465047	-0.390587
C	3.203152	-2.664547	-1.428211
C	1.911066	-2.163032	-1.323838
C	2.003245	-0.448424	2.067659
C	1.813694	1.045236	1.747924
C	0.607710	4.843064	-0.899238
H	1.735793	1.024716	-0.404447
H	0.656433	3.264393	1.106562
H	-1.360731	2.027272	0.782216
H	-6.155415	-1.968286	-0.443909
H	-4.821985	-2.990806	0.147211
H	-4.785442	-2.343506	-1.520334
H	-2.775807	-2.752193	-0.643586
H	-0.524987	-2.887084	-0.611265
H	4.413746	-1.600105	1.550442
H	1.066458	-0.852294	2.462771
H	2.748032	-0.553012	2.858561
H	1.259689	1.518200	2.566205
H	2.785469	1.542152	1.681779
H	1.444633	5.341307	-1.390468
H	-0.292700	5.064253	-1.475050
H	0.491846	5.244349	0.108155
H	5.115795	-2.854313	-0.460382
H	3.502674	-3.202868	-2.320217
H	1.214437	-2.305735	-2.142288

Compound: monomer2, Conformer: 02, Energy:-1015.889996 Hartree, Solvent: chloroform, Boltzmann %: 49.434

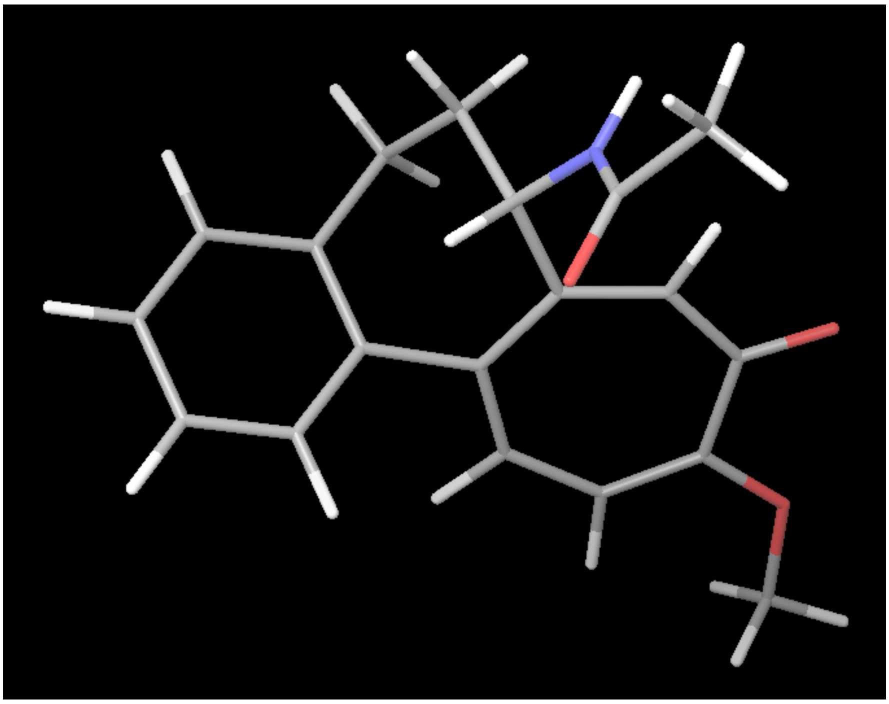

O	1.048084	2.750689	-1.981512
C	0.844599	3.347901	-0.930055
N	0.819513	2.714491	0.274167
C	1.072780	1.291865	0.419433
C	-0.173024	0.396363	0.284466
C	-1.386212	0.974084	0.522536
C	-2.762755	0.522255	0.529283
O	-3.645602	1.293430	0.927843
C	-3.148086	-0.814857	0.028989
O	-4.485189	-0.938834	-0.007791
C	-5.082130	-2.142469	-0.488548
C	-2.302841	-1.828783	-0.334939
C	-0.890654	-1.901802	-0.343852
C	0.086542	-0.974328	-0.076066
C	1.499582	-1.453832	-0.190170
C	2.420631	-1.240725	0.854304
C	3.710942	-1.754382	0.738642

C	4.107281	-2.463683	-0.390886
C	3.204082	-2.663491	-1.428412
C	1.911840	-2.162395	-1.323917
C	2.003800	-0.447711	2.067539
C	1.813636	1.045888	1.747836
C	0.604784	4.843099	-0.899293
H	1.735589	1.025356	-0.404491
H	0.655079	3.264561	1.106535
H	-1.361198	2.026805	0.782373
H	-4.783947	-2.344979	-1.520248
H	-4.821425	-2.992321	0.147251
H	-6.154571	-1.969888	-0.444613
H	-2.774727	-2.753334	-0.642716
H	-0.523849	-2.887321	-0.610912
H	4.414626	-1.598612	1.550089
H	1.067228	-0.851935	2.462787
H	2.748732	-0.551998	2.858335
H	1.259526	1.518618	2.566170
H	2.785206	1.543182	1.681614
H	-0.295974	5.063685	-1.474785
H	0.489060	5.244353	0.108120
H	1.441202	5.341851	-1.390857
H	5.116869	-2.852626	-0.460764
H	3.503689	-3.201730	-2.320436
H	1.215171	-2.305358	-2.142285

Compound: monomer2, Conformer: 03, Energy:-1015.886694 Hartree, Solvent: chloroform, Boltzmann %: 1.497

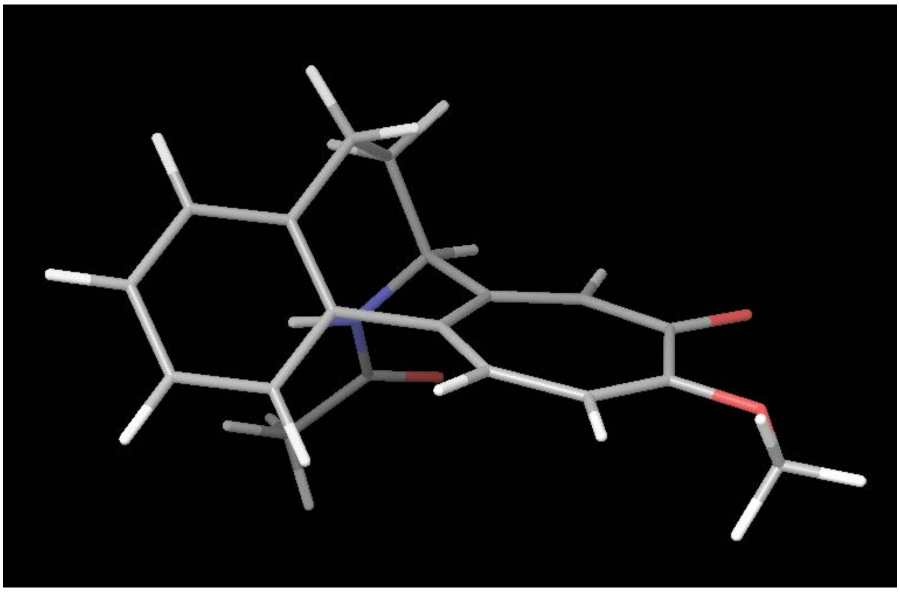

O	-1.069385	3.607774	-0.850373
C	-1.847185	2.670395	-1.000497
N	-1.831032	1.562477	-0.212484
C	-0.889585	1.398316	0.894756
C	0.352572	0.577940	0.504637
C	1.538294	1.228575	0.709982
C	2.942545	0.906865	0.537785
O	3.793627	1.712091	0.933504
C	3.384333	-0.334558	-0.133854
O	4.718643	-0.344172	-0.279177
C	5.364159	-1.442568	-0.922444
C	2.588849	-1.368938	-0.548003
C	1.189660	-1.555732	-0.464438
C	0.168565	-0.753818	-0.013807
C	-1.203062	-1.348791	-0.091258
C	-2.065424	-1.340204	1.022975
C	-3.314865	-1.952765	0.920545
C	-3.727065	-2.564319	-0.258798
C	-2.880655	-2.566761	-1.362086

C	-1.631149	-1.964887	-1.273799
C	-1.635115	-0.663729	2.298998
C	-1.577301	0.869205	2.174128
C	-2.901075	2.686523	-2.089246
H	-0.547395	2.409871	1.100981
H	-2.496766	0.828940	-0.404319
H	1.456300	2.235757	1.108383
H	5.006308	-1.563563	-1.947929
H	6.421534	-1.190373	-0.931171
H	5.212659	-2.369539	-0.363781
H	3.100496	-2.213530	-0.991928
H	0.878951	-2.533236	-0.817802
H	-3.970820	-1.955250	1.785157
H	-2.314890	-0.930363	3.110573
H	-0.645787	-1.032846	2.586378
H	-2.585641	1.290870	2.206426
H	-1.039700	1.267848	3.038257
H	-3.530381	3.567541	-1.953733
H	-2.403120	2.782894	-3.055491
H	-3.529872	1.795620	-2.099527
H	-4.702905	-3.033276	-0.314940
H	-3.191924	-3.030402	-2.291254
H	-0.981883	-1.954344	-2.141980

S10. Comparison of ^{1}H experimentally measured and DFT chemical shifts for colchicine (46mM) in chloroform using mpw1pw91/6311+G(2d,p) and B3LYP/6311+G(2d,p)

#	chloroform	mpw1pw91/6311+G(2d,p)		B3LYP/6311+G(2d,p)	
	Experimental 46mM	DFT Calculated	Deviation	DFT Calculated	Deviation
H_4	6.52	6.41	0.11	6.37	0.15
H_5	2.51	2.44	0.07	2.39	0.12
H_5	2.37	2.38	0.01	2.37	0.00
H_6	2.31	2.13	0.18	2.12	0.19
H_6	1.91	1.69	0.22	1.65	0.26
H_7	4.63	4.48	0.15	4.48	0.15
H_8	7.57	7.19	0.38	7.17	0.40
H_11	6.87	6.56	0.31	6.51	0.36
H_12	7.33	7.06	0.27	7.04	0.29
H_13	3.63	3.56	0.07	3.60	0.03
H_14	3.92	3.68	0.24	3.72	0.20
H_15	3.88	3.70	0.18	3.75	0.13
H_17	1.95	1.78	0.17	1.76	0.19
H_18	3.99	3.75	0.24	3.78	0.21
H_N	7.85	5.33	2.52	5.35	2.50
		**MAE**	**0.19**	**MAE**	**0.19**

S11. Comparison of ^{1}H experimentally measured and DFT chemical shifts for colchicine (0.46mM) in chloroform using mpw1pw91/6311+G(2d,p) and B3LYP/6311+G(2d,p)

#	chloroform	mpw1pw91/6311+G(2d,p)		B3LYP/6311+G(2d,p)	
	Experimental 0.46mM	DFT Calculated	Deviation from expt	DFT Calculated	Deviation from expt
H_4	6.52	6.41	0.11	6.37	0.15
H_5	2.52	2.44	0.08	2.39	0.13
H_5	2.41	2.38	0.03	2.37	0.04
H_6	2.22	2.13	0.09	2.12	0.10
H_6	1.76	1.69	0.07	1.65	0.11
H_7	4.63	4.48	0.15	4.48	0.15
H_8	7.34	7.19	0.15	7.17	0.17
H_11	6.79	6.56	0.23	6.51	0.28
H_12	7.27	7.06	0.21	7.04	0.23
H_13	3.63	3.56	0.07	3.60	0.03
H_14	3.93	3.68	0.25	3.72	0.21
H_15	3.89	3.70	0.19	3.75	0.14
H_17	1.99	1.78	0.21	1.76	0.23
H_18	3.98	3.75	0.23	3.78	0.20
H_N	5.89	5.33	0.56	5.35	0.54
		**MAE**	**0.15**	**MAE**	**0.15**

S12. Comparison of ^{13}C experimentally measured and DFT chemical shifts for colchicine in chloroform using mpw1pw91/6311+G(2d,p) and B3LYP/6311+G(2d,p)

#	chloroform	mpw1pw91/6311+G(2d,p)		B3LYP/6311+G(2d,p)	
	Experimental 46mM	DFT Calculated	Deviation	DFT Calculated	Deviation
C_1	151.2	150.4	0.8	151.1	0.1
C_2	141.6	140.5	1.1	141.3	0.3
C_3	153.6	152.7	0.9	153.4	0.2
C_4	107.3	104.4	2.9	103.9	3.4
C_4a	134.2	135.4	1.2	136.1	1.9
C_5	29.9	31.0	1.1	31.6	1.7
C_6	36.5	38.9	2.4	40.1	3.6
C_7	52.6	52.1	0.5	52.8	0.2
C_7a	152.3	150.6	1.7	151.1	1.2
C_8	130.5	130.9	0.4	130.4	0.1
C_9	179.5	174.5	5.0	174.4	5.1
C_10	163.8	164.2	0.4	164.8	1.0
C_11	112.8	109.0	3.8	108.6	4.2
C_12	135.6	136.6	1.0	136.4	0.8
C_12a	136.9	135.6	1.3	136.4	0.5
C_12b	125.6	126.4	0.8	127.1	1.5
C_13	61.6	58.5	3.1	58.6	3.0
C_14	61.4	58.0	3.4	58.2	3.2
C_15	56.1	52.9	3.2	53.1	3.0
C_16	170.1	166.4	3.7	166.7	3.4
C_17	22.8	21.9	0.9	21.4	1.4
C_18	56.4	53.3	3.1	53.5	2.9
		**MAE**	**1.9**	**MAE**	**1.9**

S13. Comparison of ^{1}H experimentally measured and DFT chemical shifts for colchicine (46mM) in DMSO using mpw1pw91/6311+G(2d,p) and B3LYP/6311+G(2d,p)

#	chloroform	mpw1pw91/6311+G(2d,p)		B3LYP/6311+G(2d,p)	
	Experimental 46mM	DFT Calculated	Deviation from expt	DFT Calculated	Deviation from expt
H_4	6.76	6.48	0.28	6.45	0.31
H_5	2.58	2.42	0.16	2.36	0.22
H_5	2.21	2.25	0.04	2.24	0.03
H_6	2.01	2.06	0.05	2.06	0.05
H_6	1.81	1.66	0.15	1.63	0.18
H_7	4.32	4.31	0.01	4.31	0.01
H_8	7.13	7.25	0.12	7.23	0.10
H_11	7.02	6.72	0.30	6.67	0.35
H_12	7.10	7.10	0.00	7.08	0.02
H_13	3.52	3.52	0.00	3.56	0.04
H_14	3.78	3.59	0.19	3.63	0.15
H_15	3.83	3.65	0.18	3.70	0.13
H_17	1.84	1.72	0.12	1.69	0.15
H_18	3.87	3.73	0.14	3.77	0.10
H_N	8.57	5.58	2.99	5.61	2.96
		**MAE**	**0.13**	**MAE**	**0.13**

S14. Comparison of ^{1}H experimentally measured and DFT chemical shifts for colchicine (0.46mM) in DMSO using mpw1pw91/6311+G(2d,p) and B3LYP/6311+G(2d,p)

#	chloroform	mpw1pw91/6311+G(2d,p)		B3LYP/6311+G(2d,p)	
	Experimental 0.46mM	DFT Calculated	Deviation from expt	DFT Calculated	Deviation from expt
H_4	6.76	6.48	0.28	6.447	0.31
H_5	2.52	2.42	0.10	2.361	0.16
H_5	2.21	2.25	0.04	2.243	0.03
H_6	2.00	2.06	0.06	2.057	0.06
H_6	1.81	1.66	0.15	1.627	0.18
H_7	4.32	4.31	0.01	4.308	0.01
H_8	7.13	7.25	0.12	7.228	0.10
H_11	7.02	6.72	0.30	6.672	0.35
H_12	7.10	7.10	0.00	7.079	0.02
H_13	3.51	3.52	0.01	3.558	0.05
H_14	3.78	3.59	0.19	3.63	0.15
H_15	3.83	3.65	0.18	3.702	0.13
H_17	1.84	1.72	0.12	1.693	0.15
H_18	3.87	3.73	0.14	3.765	0.10
H_N	8.55	5.58	2.97	5.607	2.94
		**MAE**	**0.12**	**MAE**	**0.13**

S15. Comparison of ^{13}C experimentally measured and DFT chemical shifts for colchicine (46mM) using mpw1pw91/6311+G(2d,p) and B3LYP/6311+G(2d,p)

#	chloroform	mpw1pw91/6311+G(2d,p)		B3LYP/6311+G(2d,p)	
	Experimental 46mM	DFT Calculated	Deviation from expt	DFT Calculated	Deviation from expt
C_1	151.2	148.9	2.3	149.6	1.6
C_2	141.6	139.1	2.5	140.0	1.6
C_3	153.6	151.8	1.8	152.5	1.1
C_4	107.3	104.5	2.8	104.0	3.3
C_4a	134.2	135.1	0.9	135.8	1.6
C_5	29.9	30.4	0.5	31.0	1.1
C_6	36.5	38.2	1.7	39.3	2.8
C_7	52.6	52.2	0.4	53.0	0.4
C_7a	152.3	150.4	1.9	150.8	1.5
C_8	130.5	129.6	0.9	129.1	1.4
C_9	179.5	173.8	5.7	173.7	5.8
C_10	163.8	163.0	0.8	163.4	0.4
C_11	112.8	109.5	3.3	109.2	3.6
C_12	135.6	136.2	0.6	135.9	0.3
C_12a	136.9	135.4	1.5	136.2	0.7
C_12b	125.6	124.9	0.7	125.6	0.0
C_13	61.6	58.0	3.6	58.1	3.5
C_14	61.4	57.9	3.5	58.1	3.3
C_15	56.1	52.9	3.2	53.2	2.9
C_16	170.1	166.7	3.4	166.9	3.2
C_17	22.8	21.8	1.0	21.4	1.4
C_18	56.4	53.3	3.1	53.5	2.9
		**MAE**	**2.1**	**MAE**	**2.0**

S16. Optimized coordinates for the dimer of the cutdown colchicine (dimer 2) in chloroform solvent (wB97XD/6-311+g(2d,p))

Conformational search was performed using MacroModel as described above.

All conformers were optimized with Gaussian 09 using B3LYP/631G(d) in vacuum and the vibrational frequencies where checked for a true minimum. This resulted in a single unique conformer for colchicine dimer. The unique conformation was further optimized with wB97XD/6-311+g(2d,p) and a Polarizable Continuum Model (PCM) for chloroform and the vibrational frequencies where checked again for a true minimum.

NMR parameters (nmr=giao) were calculated with a single-point calculation, using two functional and basis set combinations; b3lyp/6-311+g(2d,p), mpw1pw91/6-311+g(2d,p) and wB97XD/6-311+g(2d,p) using the optimized structures from the wB97XD/6-311+g(2d,p) calculation. The integrated equation formalism polarized continuum model (IEFPCM) for chloroform were used in all NMR calculations The computed NMR shielding tensors were converted to chemical shifts by the approach of using empirical scaling factors that are derived from linear regression analysis of a test set of molecules at the same level of theory.

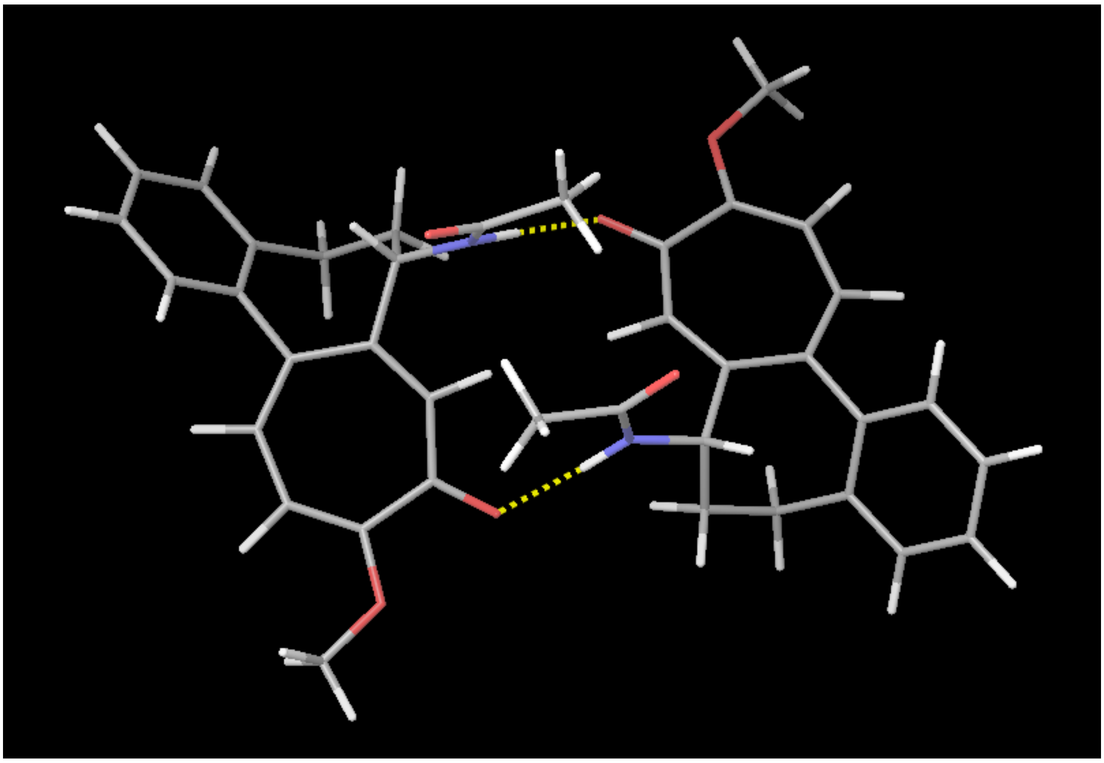

Compound: dimer2, Conformer: cutdown, Energy:-2031.096429 Hartree, Solvent: chloroform, Boltzmann %: 100

O	-2.253086	-4.617238	-0.897195
O	-0.520260	-2.783163	-0.711745
O	-2.210580	0.905071	2.892706
N	-1.593186	1.553043	0.819748
C	-2.766703	-3.399195	-0.706821
C	-1.690599	-2.401621	-0.576013
C	-1.946254	-1.021410	-0.255444
C	-3.062130	-0.257853	-0.112257
C	-2.859999	1.226312	0.214068
C	-3.039020	2.095911	-1.039612
C	-4.290678	1.738727	-1.850451
C	-5.478685	1.497656	-0.958938
C	-6.524259	2.408483	-0.881062
C	-7.594558	2.199025	-0.021890
C	-7.624304	1.066550	0.777046
C	-6.587931	0.147288	0.705768

C	-5.514648	0.347481	-0.158017
C	-4.829787	-1.958692	-0.487922
C	-4.428465	-0.674824	-0.258124
C	-4.108201	-3.162902	-0.655880
C	-3.113754	-5.734440	-1.026111
C	-1.385864	1.423089	2.148389
C	-0.061187	1.936790	2.659298
H	-0.876760	2.000481	0.248186
H	-1.010074	-0.494335	-0.104390
H	-3.634326	1.486955	0.939155
H	-3.088612	3.137023	-0.710084
H	-2.152954	1.997690	-1.673971
H	-4.093838	0.841197	-2.444565
H	-4.504082	2.543163	-2.556042
H	-6.499130	3.296265	-1.505251
H	-5.904181	-2.095764	-0.565432
H	-4.750664	-4.024394	-0.789842
H	-2.459742	-6.593861	-1.150891
H	-3.758965	-5.636622	-1.903674
H	-3.724726	-5.867501	-0.128886
H	0.399658	2.651058	1.977500
H	0.625348	1.098892	2.804635
H	-0.223263	2.404251	3.629824
O	2.253065	4.617245	-0.897169
O	0.520255	2.783148	-0.711797
O	2.210611	-0.905121	2.892729
N	1.593185	-1.553025	0.819758
C	2.766692	3.399203	-0.706812
C	1.690595	2.401620	-0.576027
C	1.946254	1.021413	-0.255448
C	3.062131	0.257858	-0.112262

C	2.859998	-1.226304	0.214074
C	3.039017	-2.095910	-1.039603
C	4.290669	-1.738725	-1.850452
C	5.478682	-1.497648	-0.958949
C	6.524258	-2.408474	-0.881078
C	7.594563	-2.199012	-0.021914
C	7.624315	-1.066533	0.777016
C	6.587940	-0.147274	0.705743
C	5.514650	-0.347471	-0.158032
C	4.829782	1.958702	-0.487927
C	4.428465	0.674832	-0.258133
C	4.108191	3.162912	-0.655872
C	3.113715	5.734465	-1.026048
C	1.385889	-1.423125	2.148410
C	0.061221	-1.936849	2.659320
H	0.876764	-2.000484	0.248205
H	1.010076	0.494335	-0.104391
H	3.634326	-1.486946	0.939161
H	3.088614	-3.137019	-0.710070
H	2.152946	-1.997695	-1.673957
H	4.093823	-0.841198	-2.444567
H	4.504070	-2.543163	-2.556042
H	6.499124	-3.296260	-1.505262
H	5.904175	2.095778	-0.565442
H	4.750654	4.024406	-0.789825
H	2.459689	6.593879	-1.150801
H	3.758929	5.636687	-1.903613
H	3.724684	5.867508	-0.128819
H	-0.399559	-2.651201	1.977565
H	-0.625366	-1.098974	2.804549
H	0.223284	-2.404210	3.629895

H	-8.400242	2.923047	0.026195
H	-8.448665	0.899460	1.460920
H	-6.601503	-0.731423	1.342025
H	8.400248	-2.923034	0.026168
H	8.448680	-0.899441	1.460883
H	6.601516	0.731440	1.341996

S17. Comparison of ^{1}H experimentally measured and DFT calculated chemical shifts for cut-down colchicine dimer 2.

^{1}H #	Expt (46 mM)	Calculated NMR Chemical shifts (functional/basis set)		
		mpw1pw91/6-311+g(2d,p)	b3lyp/6-311+g(2d,p)	wB97XD/6-311+g(2d,p)
H-7	4.63	4.26	4.23	4.25
H-8	7.57	7.84	7.80	8.01
H-11	6.87	6.79	6.75	6.67
H-12	7.33	7.23	7.20	7.28
H-17	1.95	1.60	1.56	1.68
H-18	3.99	8.78	3.83	3.78
NH	7.85	9.22	9.31	9.32
